# Increased intratumoral mast cells foster immune suppression and gastric cancer progression through TNF-α-PD-L1 pathway

**DOI:** 10.1186/s40425-019-0530-3

**Published:** 2019-02-26

**Authors:** Yipin Lv, Yongliang Zhao, Xianhua Wang, Na Chen, Fangyuan Mao, Yongsheng Teng, Tingting Wang, Liusheng Peng, Jinyu Zhang, Ping Cheng, Yugang Liu, Hui Kong, Weisan Chen, Chuanjie Hao, Bin Han, Qiang Ma, Quanming Zou, Jun Chen, Yuan Zhuang

**Affiliations:** 1 National Engineering Research Center of Immunological Products, Department of Microbiology and Biochemical Pharmacy, College of Pharmacy, Third Military Medical University, No.30 Gaotanyan Street, Chongqing, 400038 China; 2 Department of General Surgery and Centre of Minimal Invasive Gastrointestinal Surgery, Southwest Hospital, Third Military Medical University, No.30 Gaotanyan Street, Chongqing, 400038 China; 3 Department of Obstetrics and Gynecology, Research Institute of Surgery, Daping Hospital, Third Military Medical University, Chongqing, China; 4 La Trobe Institute of Molecular Science, School of Molecular Science, La Trobe University, Bundoora, Vic 3085 Australia; 5 Affiliated Hospital of North Sichuan Medical College, Nanchong, Sichuan Province China

**Keywords:** Gastric cancer, Tumor microenvironment, Mast cells, TNF-α, PD-L1, Immunotherapy

## Abstract

**Background:**

Mast cells are prominent components of solid tumors and exhibit distinct phenotypes in different tumor microenvironments. However, the nature, regulation, function, and clinical relevance of mast cells in human gastric cancer (GC) are presently unknown.

**Methods:**

Flow cytometry analyses were performed to examine level and phenotype of mast cells in samples from 114 patients with GC. Multivariate analysis of prognostic factors for overall survival was performed using the Cox proportional hazards model. Kaplan-Meier plots for patient survival were performed using the log-rank test. Mast cells, T cells and tumor cells were isolated or generated, stimulated and/or cultured for in vitro and in vivo function assays.

**Results:**

Patients with GC showed a significantly higher mast cell infiltration in tumors. Mast cell levels increased with tumor progression and independently predicted reduced overall survival. These tumor-infiltrating mast cells accumulated in tumors by CXCL12-CXCR4 chemotaxis. Intratumoral mast cells expressed higher immunosuppressive molecule programmed death-ligand 1 (PD-L1), and mast cells induced by tumors strongly express PD-L1 proteins in both time-dependent and dose-dependent manners. Significant correlations were found between the levels of PD-L1^+^ mast cells and pro-inflammatory cytokine TNF-α in GC tumors, and tumor-derived TNF-α activated NF-κB signaling pathway to induce mast cell expression of PD-L1. The tumor-infiltrating and tumor-conditioned mast cells effectively suppressed normal T-cell immunity through PD-L1 in vitro, and tumor-conditioned mast cells contributed to the suppression of T-cell immunity and the growth of human GC tumors in vivo; the effect could be reversed by blocking PD-L1 on these mast cells.

**Conclusion:**

Thus, our results illuminate novel immunosuppressive and protumorigenic roles of mast cells in GC, and also present a novel mechanism in which PD-L1 expressing mast cells link the proinflammatory response to immune tolerance in the GC tumor milieu.

**Electronic supplementary material:**

The online version of this article (10.1186/s40425-019-0530-3) contains supplementary material, which is available to authorized users.

## Background

Gastric cancer (GC), as a severe health problem, has been the fourth most common malignancies and the second leading cause of cancer death worldwide [1]. In some low/middle income countries with poor sanitation or high risk of *helicobacter pylori* infection, it has been one of the major causes of cancer death [2, 3]. Despite significant progress made in prevention, diagnose, and therapeutic options in recent years [4, 5], many questions remain unanswered, especially the pathogenesis of GC. Nowadays, it is generally believed that the development and prognosis of GC are influenced by the cross-talk between tumors and host immune system [6, 7]. Previous studies have focused on the crucial role for adaptive immunity in determining the clinical outcomes of GC patients [8]. However, little is known about the role of innate immunity and innate immune cells during GC development and progression.

Mast cells are a group of innate immune cells, which have profound immunomodulatory effects on tumor progression [9, 10], such as angiogenesis [11], tumor microenvironment reconstruction [12] and interaction with other immune cells [13]. At present, limited studies on mast cells in GC mainly focus on the correlation between the survival rate of GC patients and their GC mast cell infiltration by immunohistochemistry [14], and a few on the relationship between infiltrated mast cell density and local angiogenesis [15]. Overall, these studies suggest that mast cells may be a therapeutic target for GC. However, the phenotype, functional regulation and clinical correlation of mast cells in human GC microenvironment remain unclear.

Herein, we investigate the interplays among mast cells, T cells and tumor cells in the GC microenvironment. We show that mast cells could be recruited to tumor microenvironment through CXCL12-CXCR4 chemotaxis axis. Moreover, tumor-derived TNF-α efficiently induces programmed death-ligand 1 (PD-L1) expression on mast cells by activating nuclear factor kappa-light-chain-enhancer of activated B cells (NF-κB) signaling pathways. In turn, these mast cells inhibit the normal function of T cells in a PD-L1-dependent manner, which could suppress antitumor immunity in GC.

Our data suggest a protumorigenic role of mast cells with an immunosuppressive phenotype in GC. These tumor-infiltrating mast cells increase with tumor progression and are negatively correlated with patient survival after surgery, suggesting that these mast cells may be a novel target in novel GC therapy.

## Methods

### Patients and specimens

Fresh gastric tumor (homogeneous cellularity, without foci of necrosis, including intratumoral and marginal tissues), peritumoral and non-tumor (non-tumor tissues, at least 5 cm distant from the tumor site) tissues and autologous peripheral blood were obtained from patients with GC who underwent surgical resection at the Southwest Hospital of Third Military Medical University. None of the patients had received chemotherapy or radiation before the sample was taken. Patients with infectious diseases, autoimmune diseases or multiple primary cancers were excluded. The clinical stage of tumors was determined according to the TNM classification system of the International Union Against Cancer (7th edition). The study was approved by the Ethics Committee of the Southwest Hospital of Third Military Medical University. Each subject provided written informed consent. Additional file 1: Table S1 lists antibodies and other reagents.

### Isolation of single cells from GC tissues

Fresh tissues were washed 3 times with Hank’s solution containing 1% FCS and cut into small pieces. Specimens were collected in RPMI 1640 medium containing collagenase IV (1 mg/ml) and deoxyribonuclease I (10 mg/ml) and mechanically separated using the gentle MACS Dissociator (Miltenyi Biotec). Dissociated cell suspensions were further incubated for 1 h under continuous rotation at 37 °C. The cell suspensions were then filtered by a 70 μm cell filter (BD Labware). Cell viability, as measured by trypan blue exclusion staining, was typically > 90%.

### Isolation of mast cells and T cells

As mentioned above, tumor and non-tumor tissues were treated as single cell suspension. Then the single cell suspension was stained with anti-human CD45, anti-human CD117 and anti-human FcεRI antibodies, and mast cells from autologous tumor and non-tumor tissues were sorted by fluorescence activating cell sorter (FACS) (FACSAria II; BD Biosciences). Density gradient centrifugation was used to isolate peripheral blood mononuclear cells (PBMCs) from autologous GC patients and healthy donors by using Ficoll-Paque Plus. CD3^+^ T cells from PBMCs were purified with CD3 microbeads. The sorted cells were used only when their viability was determined > 90% and their purity was determined > 95%.

### Preparation of TTCS and NTCS and supernatant-conditioned mast cells

Tumor tissue culture supernatants (TTCS) or non-tumor tissue culture supernatants (NTCS) were prepared by plating autologous tumor or non-tumor gastric tissues in 1 ml RPMI 1640 medium for 24 h. The supernatant was then harvested by centrifugation. To generate supernatant-conditioned mast cells, primary human umbilical cord blood-derived cultured mast cells (hCBMCs) or LAD2 cells were first harvested and cultured with autologous 50% TTCS or NTCS for 24 h, and then washed with RPMI-1640 medium for 3 times.

### Chemotaxis assay

Fluorescence-activated cell sorter sorted tumor-infiltrating mast cells (1 × 10^5^) from fresh human tumor tissues were transferred into the upper chambers of 8-μm pore size Transwells (Corning). Autologous 50% TTCS or NTCS as the sources of chemoattractants were placed in the lower chambers. After 24-h culture at 37 °C, migration was quantified by counting cells in the lower chamber and cells adhering to the bottom of the membrane. In some cases, blocking antibody for CXCR4 (20 μg/ml, IgG2b) or control IgG2b was added into mast cell suspensions and incubated for 2 h before chemotaxis assay. Furthermore, CXCL12 neutralizing antibody (20 μg/ml, IgG1) or control IgG1 was added into TTCS in some assays. RPMI-1640 medium and chemokine CXCL12 (100 ng/ml) were placed in the lower chambers as blank and positive controls respectively.

### Mast cell stimulation

The hCBMCs, LAD2 cells, HMC-1 cells were stimulated with 50% TTCS or 50% autologous NTCS, or 50% TTCS for 3, 6 or 12 h, or with different concentrations TTCS (20, 40, 80%) for 24 h, or with 50% TTCS together with a neutralizing antibody against human TNF-α (20 μg/ml) for 24 h, or with human recombinant (hr) cytokines (100 ng/ml) for 24 h. After stimulation, the cells were harvested for flow cytometric analysis and western blot. For the signaling pathway inhibition experiments, the cells were pretreated with 5 μl (10 μM) BAY 11–7082 (an IκBα inhibitor), U0126 (a MEK1/2 inhibitor), SP600125 (a c-Jun N-terminal kinase (JNK) inhibitor), SB203580 (a mitogen-activated protein kinase (MAPK) inhibitor) or Wortmannin (a PI3K inhibitor) for 1 h, then were stimulated with 50% TTCS or hr. TNF-α (100 ng/ml) for 24 h and harvested as above. As the inhibitors were dissolved in dimethyl sulfoxide (DMSO), parallel cell groups were treated with DMSO (5 μl) or culture media as controls.

### In vitro mast cell-T cell co-culture system

In a 5-day incubation, magnetic bead-purified peripheral CD3^+^ T cells (2 × 10^5^ cells/well in 96-well plates) were labeled with carboxyfluorescein succinimidyl ester (CFSE) and co-cultured with autologous mast cells isolated from tumor or non-tumor tissues at a 2:1 (T cell: mast cell) ratio in 200 μl RPMI-1640 medium containing 10% fatal bovine serum (FBS), rh IL-2 (20 IU/ml), anti-CD3 (2 μg/ml), and anti-CD28 (1 μg/ml) antibodies, with or without a human PD-L1 neutralizing antibody (20 μg/ml). In another co-culture system, CFSE-labeled magnetic bead-purified peripheral CD3^+^ T cells (2 × 10^5^ cells/well in 96-well plates) were co-cultured with TTCS- or NTCS-conditioned hCBMCs or LAD2 cells at a 2:1 ratio as described above, in the presence or absence of a neutralizing antibody against human PD-L1 (20 μg/ml). After 5-day incubation, the supernatants were harvested for ELISA and the cells were harvested for intracellular cytokine staining.

### In vivo tumor inhibition assay

All animal experiments were undertaken with the approval from the Animal Ethical and Experimental Committee of Third Military Medical University. 10^6^ GC cells (SGC-7901 cells) in 100 μl of buffered saline were subcutaneously injected into the axillary tissues of female nonobese diabetic/severe combined immunodeficiency (NOD/SCID) mice (5–7 week, one tumor per mouse). The hCBMCs (referred to as mast cells) were stimulated with 50% TTCS for 24 h. Then, 5 × 10^6^ anti-CD3- and anti-CD28-stimulated (2 μg/ml anti-CD3 and 1 μg/ml anti-CD28) polyclonal T cells were co-cultured with mast cells, or TTCS-conditioned mast cells (TCM) at a 2:1 ratio in the presence or absence of a neutralizing antibody against human PD-L1 (20 μg/ml) or a control IgG (20 μg/ml) for 5 days, and were subsequently injected into the peritoneum in 200 μl of buffered saline on day 7 after inoculation. Tumor size was measured every 2 days by two independent observers using calipers fitted with a vernier scale. Tumor volumes (V) were calculated with the formula: V = A × B^2^/2 (A = axial diameter; B = rotational diameter). Once the mice were sacrificed, tumors were weighed and photographed, and were further fixed for immunohistochemical staining, ELISA or real-time PCR, and the spleens were dissociated into single cells for flow cytometry.

### Statistical analysis

Results are expressed as mean ± SEM. Student *t* test was generally used to analyze the differences between two groups, but when the variances differed, the Mann-Whitney U test was used. Correlations between parameters were assessed using the Pearson correlation analysis and linear regression analysis as appropriate. Overall/disease-free survival was defined as the interval between surgery and death/recurrence or between surgery and the last observation for surviving/disease-free patients. The known tumor-unrelated deaths (eg, accidental death) were excluded from the death record for this study. Cumulative survival time was calculated by the Kaplan-Meier method, and survival was measured in month; the log-rank test was applied to compare between 2 groups. Multivariate analysis of prognostic factors for patient overall survival was performed using the Cox proportional hazards model. SPSS statistical software (version 13.0) was used for all statistical analysis. All data were analyzed using 2-tailed tests, and *P* < 0.05 was considered statistically significant.

## Results

### Mast cells are enriched in GC as tumor progress and independently predict poor patient survival

**Fig. 1:**
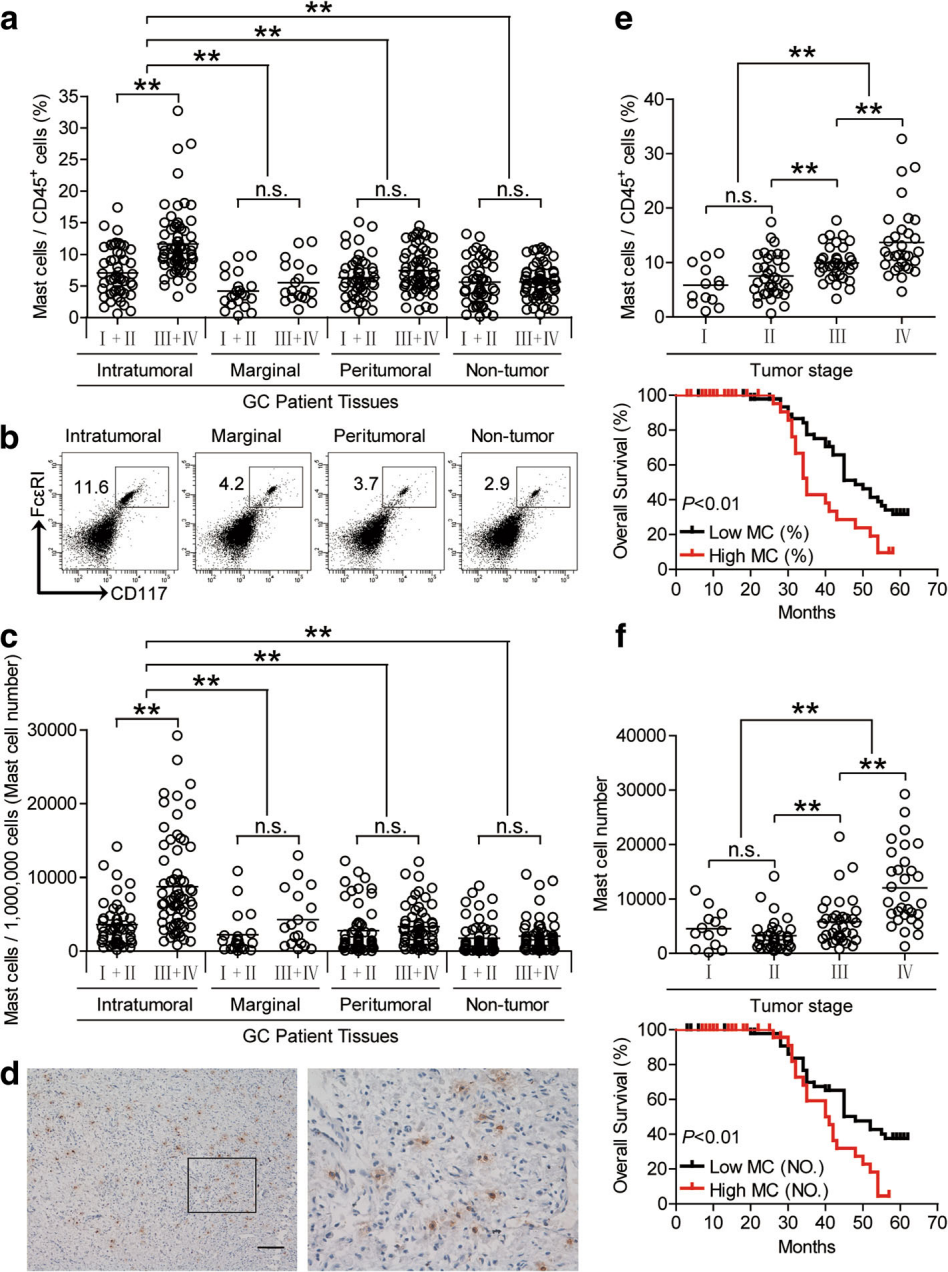
Mast cells accumulate in GC tumors with disease progression and predict poor patient survival. **a** Mast cell percentage in CD45^+^ cells or (**c**) the total number of mast cells per million total cells among TNM stages (I + II vs III + IV) in each tissue of patients with GC by gating on CD45^+^CD117^+^FcεRI^+^ cells or counting. Cumulative results from 114 GC patients were shown. **b** Dot plots of surface molecule staining for mast cells gating on CD45^+^ cells. **d** Representative analysis of tryptase^+^ (brown) mast cell distributions in tumor tissues of GC patients by immunohistochemical staining. Scale bars: 100 μm. **e** and **f** Intratumoral mast cell percentage (**e**) or mast cell number (**f**) among TNM stages was compared. Kaplan-Meier plots for overall survival by median mast cell percentage (9.315%) (**e**) or median mast cell number (4749 per million) (**f**). The horizontal bars in panels **a**, **c**, **e** and **f** represent mean values. Each ring in panels **a**, **c**, **e** and **f** represents 1 patient. **, *P* < 0.01; n.s., *P* > 0.05 for groups connected by horizontal lines. MC (%), mast cell percentage; MC (NO.), mast cell number

To evaluate the potential role of mast cells in human GC, we analyzed mast cell percentage within the total CD45^+^ leukocytes from intratumoral, marginal, peritumoral, and non-tumor tissues of GC patients at various stages. Notably, patients with GC showed a higher mast cell percentage in intratumoral tissues than marginal, peritumoral, and non-tumor tissues (Fig. 1a and b). Moreover, as the cancer progressed, the percentage of intratumoral mast cells increased significantly (Fig. 1a), and such intratumoral mast cell accumulation was most notable from stage II onwards (Fig. 1e). Similar observations were made when analyzing the total number of mast cells per million total cells in each tissue (Fig. 1c and f). Furthermore, immunohistochemical staining also showed that mast cells were accumulated in tumors (Fig. 1d), indicating a potential role for mast cells in the GC microenvironment. In keeping with this finding, increased mast cell percentage and mast cell number were correlated with increased advanced lymphatic invasion, tumor size and tumor stage (Additional file 2: Figure S1).

Next, we evaluated the clinical relevance of intratumoral mast cells in GC. Comparing patients with high (≥9.315% median level) versus low (< 9.315%) mast cell percentage level, the 62-month overall survival rates were significantly lower for those within the higher mast cell percentage group (Fig. 1e). Similar results were obtained when the patient cohort was stratified based on intratumoral mast cell number (Fig. 1f). Importantly, this finding that intratumoral mast cell percentage and/or number independently predicted survival was verified by univariate and multivariate analyses using a Cox proportional hazard model (Additional file 3: Table S3). The clinical characteristics of all the GC patients are described in Additional file 4: Table S2, and correlations between intratumoral mast cell percentage or mast cell number and clinical characteristics of GC patients are listed in Additional file 5: Table S4 and Additional file 6: Table S5. Taken together, these findings suggest that increased intratumoral mast cells are associated with tumor progression and poor survival of GC patients.

### Increased mast cell accumulation in GC is promoted by CXCL12-CXCR4-mediated chemotaxis

**Fig. 2:**
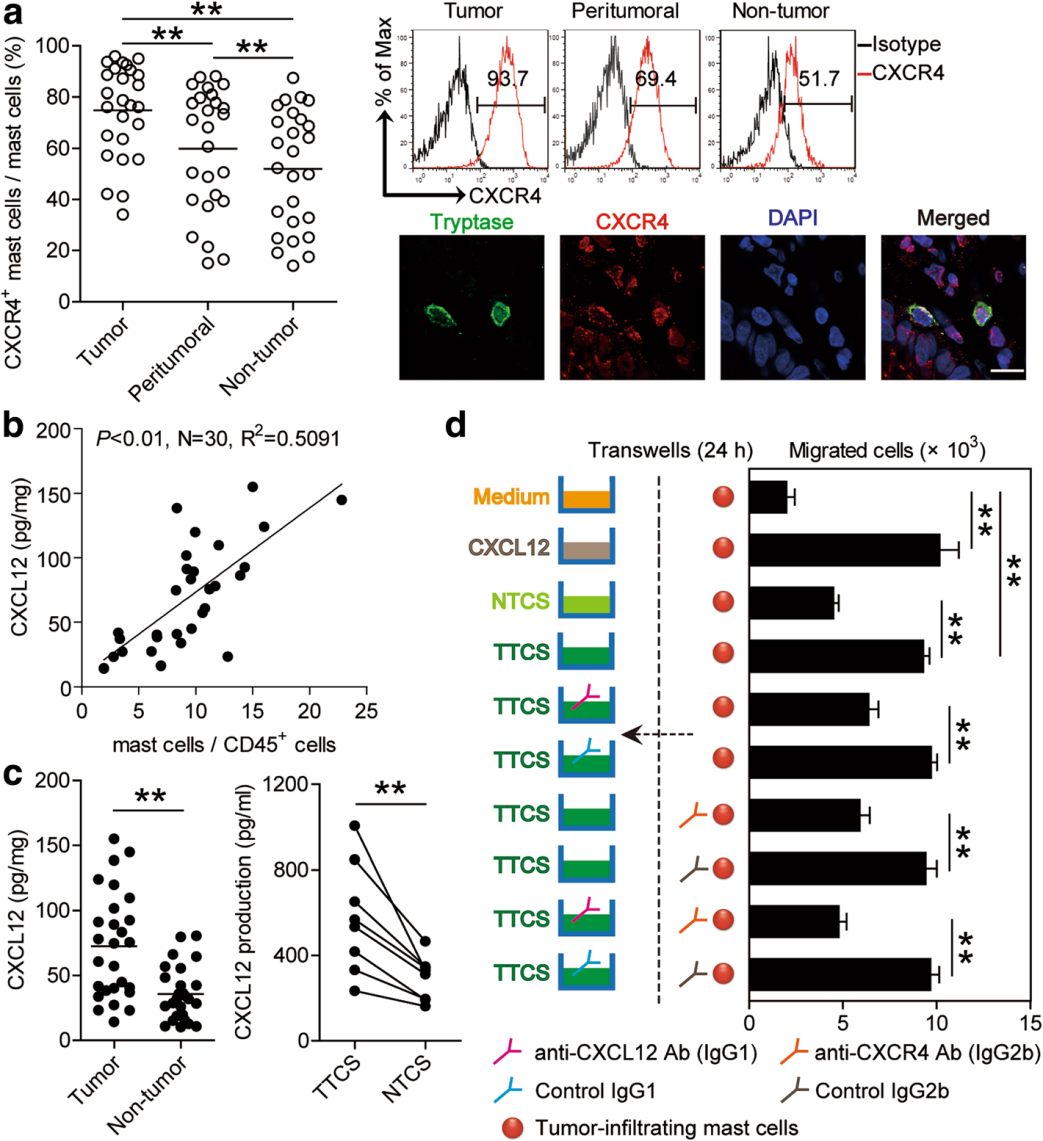
CXCL12-CXCR4 chemotaxis mediates mast cell migration and accumulation in GC tumors. **a** Statistics analysis of CXCR4^+^ mast cell percentage in total mast cells in each samples of patients with GC (*n* = 26). Expression of molecule CXCR4 on mast cells by gating on CD45^+^CD117^+^FcεRI^+^ cells. Color histograms represent staining of CXCR4; black, isotype control. Tumor-infiltrating CXCR4^+^tryptase^+^ mast cells were defined by immunofluorescence staining. Green, Tryptase; red, CXCR4; and blue, DAPI-stained nuclei. Scale bars: 10 μm. **b** The correlations between mast cells and CXCL12 production in GC tumors were analyzed. Results were expressed as percentage of mast cells in CD45^+^ cells and CXCL12 concentration in tumor tissues of patients with GC. **c** CXCL12 concentration between autologous tumor and non-tumor tissues (*n* = 27) or between autologous TTCS and NTCS (*n* = 8) was analyzed. **d** Migration of tumor-infiltrating mast cells was assessed by Transwell assay as described in Materials and methods and statistically analyzed (*n* = 3). The horizontal bars in panels **a** and **c** represent mean values. Each ring or dot in panels **a**, **b** or **c** represents 1 patient. *, *P* < 0.05; **, *P* < 0.01 for groups connected by horizontal lines

The results described above suggested that GC microenvironment triggers mast cell accumulation, so we wondered the cause of such accumulation. We first found that intratumoral mast cells expressed little Ki-67, suggesting that these cells in GC were not actively proliferative (Additional file 7: Figure S2a and b). Next, we hypothesized that GC microenvironment might induce mast cell migration into the tumors by chemotaxis. We therefore examined the chemokine receptors that are involved in myeloid cell migration. The majority of tumor-infiltrating mast cells expressed CXCR4 but not CCR2, CCR4, CCR5, CCR7, CXCR1, CXCR2 or CXCR7 (Fig. 2a; Additional file 7: Figure S2c), while, mast cells in peritumoral or non-tumor tissues showed lower CXCR4 expression (Fig. 2a). In support of this, dual immunofluorescence staining showed that tryptase^+^ mast cells expressed CXCR4 in GC tumors (Fig. 2a). Taken together, these results suggest that mast cells may be induced to migrate into the tumor microenvironment through CXCR4-mediated chemotaxis.

Interestingly, we further found that the frequency of mast cells was positively correlated with CXCL12 production (Fig. 2b), which most likely derived from EpCam^+^ tumor cells (Additional file 7: Figure S2d) in the GC microenvironment. Meanwhile, we found that the concentrations of CXCL12 in tumor tissues or tumor tissue culture supernatants (TTCS) were significantly increased when compared to that in non-tumor tissues or non-tumor tissue culture supernatants (NTCS) (Fig. 2c). To substantiate the functional significance of CXCL12-CXCR4 in the recruitment of mast cells, mast cell chemotaxis assay was performed and showed that TTCS induced significantly more tumor-infiltrating mast cell migration than NTCS from the same GC patients, and this effect was lost upon pre-treatment with neutralizing antibodies against CXCL12 and/or CXCR4 (Fig. 2d). Taken together, these data support a model wherein GC tumors secrete chemokine CXCL12 which in turn recruits mast cells into the tumor microenvironment by CXCL12-CXCR4 interaction.

### Tumor-derived factor TNF-α induces mast cells to express PD-L1

**Fig. 3:**
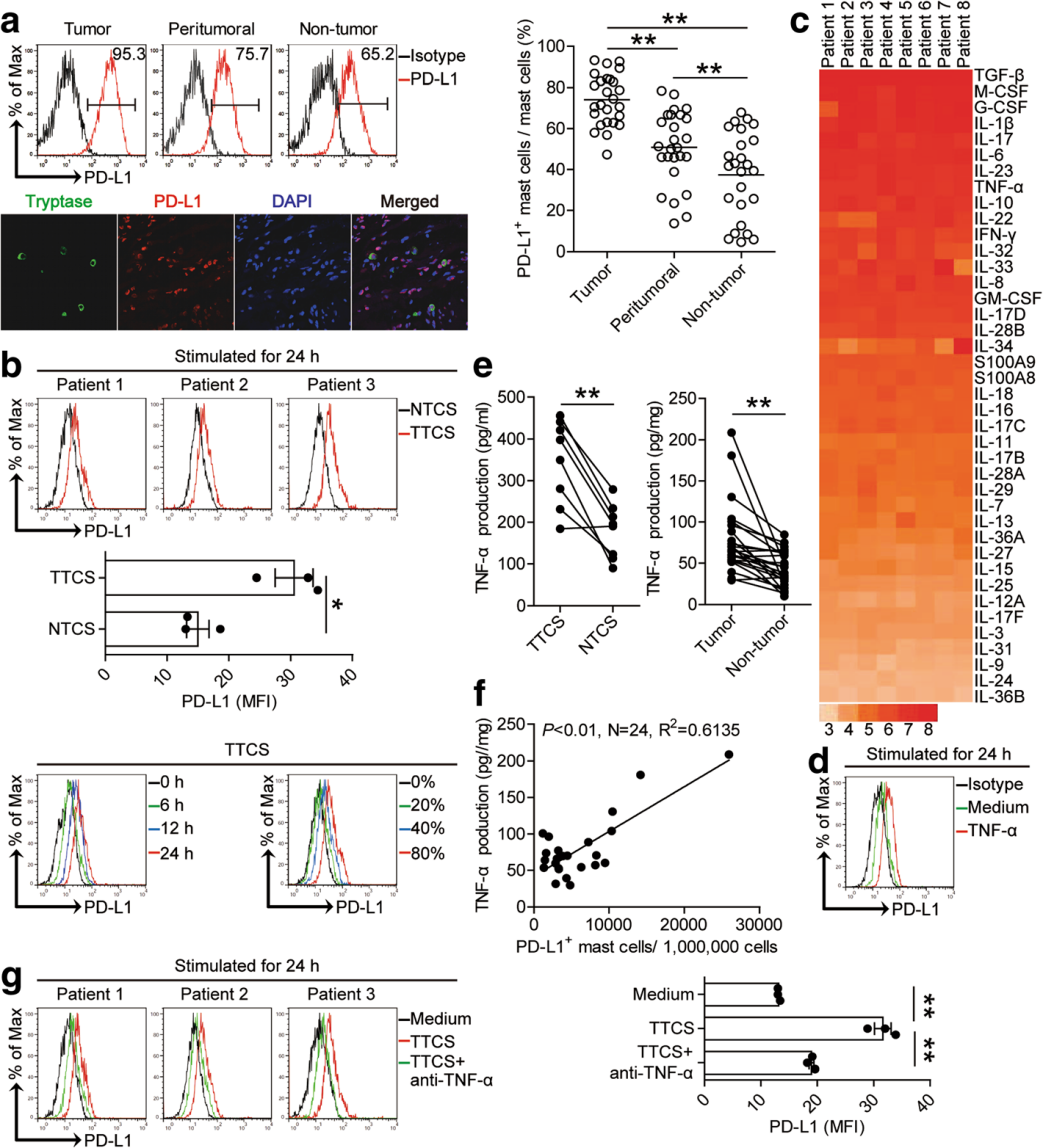
Tumor-derived factor TNF-α induces mast cells to express PD-L1. **a** Statistics analysis of PD-L1^+^ mast cell percentage in total mast cells in each samples of patients with GC (*n* = 26). Expression of molecule PD-L1 on mast cells by gating on CD45^+^CD117^+^FcεRI^+^ cells. Color histograms represent staining of PD-L1; black, isotype control. Tumor-infiltrating PD-L1^+^tryptase^+^ mast cells were defined by immunofluorescence staining. Green, Tryptase; red, PD-L1; and blue, DAPI-stained nuclei. Scale bars: 50 μm. **b** Expression of PD-L1 on hCBMCs exposed to 50% autologous TTCS and NTCS for 24 h, or exposed to 50% TTCS for 6, 12, 24 h, or exposed to 20, 40, 80% TTCS for 24 h. black, isotype control. **c** Clustering of microarray data for the expression of 40 pro-inflammatory cytokine genes in human tumor tissues from 8 GC patients. **d** Expression of PD-L1 on hCBMCs exposed to TNF-α for 24 h. black, isotype control. **e** TNF-α concentration between autologous tumor and non-tumor tissues (*n* = 24) or between autologous TTCS and NTCS (*n* = 8) was analyzed. **f** The correlations between TNF-α and PD-L1^+^ mast cells in human tumors were analyzed. Results are expressed as the number of PD-L1^+^ mast cells per million total cells and TNF-α concentration in tumor tissues. **g** Expression of PD-L1 on hCBMCs exposed to TTCS with anti-TNF-α antibody for 24 h. Each ring or dot in panels **a** and **f** represents 1 patient. *, *P* < 0.05, **, *P* < 0.01 for groups connected by horizontal lines

To better understand these intratumoral mast cells’ likely function, we performed a detailed immune-phenotype. Notably, we found that intratumoral mast cells expressed significantly higher level of immunosuppressive molecule PD-L1 (Fig. 3a) but not other molecules with immunosuppressive potential such as 2B4, glactin-3, CTLA-4, or ICOSL (Additional file 8: Figure S3a) than that expressed on peritumoral and non-tumor mast cells, indicating a potential role for PD-L1 on mast cells in the GC microenvironment. Meanwhile, we hypothesized that GC environments contribute to the immunosuppressive phenotype of mast cells. Consistent with our hypothesis, compared to NTCS-conditioned mast cells, TTCS-conditioned mast cells significantly up-regulated PD-L1 expression in both time-dependent and dose-dependent manners (Fig. 3b).

Tumor microenvironment can possess various soluble factors [16], including pro-inflammatory cytokines. To see which cytokines might induce PD-L1 expression on mast cells, we first screened pro-inflammatory cytokines in human GC environments by microarray (Fig. 3c), and stimulated normal mast cells with highly-expressed cytokines including IL-1β, IL-6, IL-10, IL-17, IL-22, IL-23, M-CSF, G-CSF, TNF-α, IFN-γ, TGF-β, etc. We found that only TNF-α remarkably up-regulated the expression of PD-L1 on mast cells (Fig. 3d; Additional file 8: Figure S3b). Next, we found that the concentrations of TNF-α in tumor tissues or TTSC were significantly increased when compared to that in non-tumor tissues or NTCS (Fig. 3e). There was clearly a positive correlation between PD-L1^+^ mast cell infiltration and TNF-α production (Fig. 3f) and a high expression of TNF-α receptor II (TNFRII) on mast cells (Additional file 8: Figure S3c) within tumors. Next, to evaluate the potential role of GC tumor-derived TNF-α in PD-L1 induction on mast cells, we added neutralizing antibody against TNF-α into TTCS/mast cell co-culture. Interestingly, antibody blockade of TNF-α efficiently inhibited the induction of PD-L1 on mast cells (Fig. 3g). These findings show that tumor-derived TNF-α plays an essential role in mast cell PD-L1 induction.

### Tumor-derived TNF-α activates NF-κB pathway to induce PD-L1 expression on mast cells

**Fig. 4:**
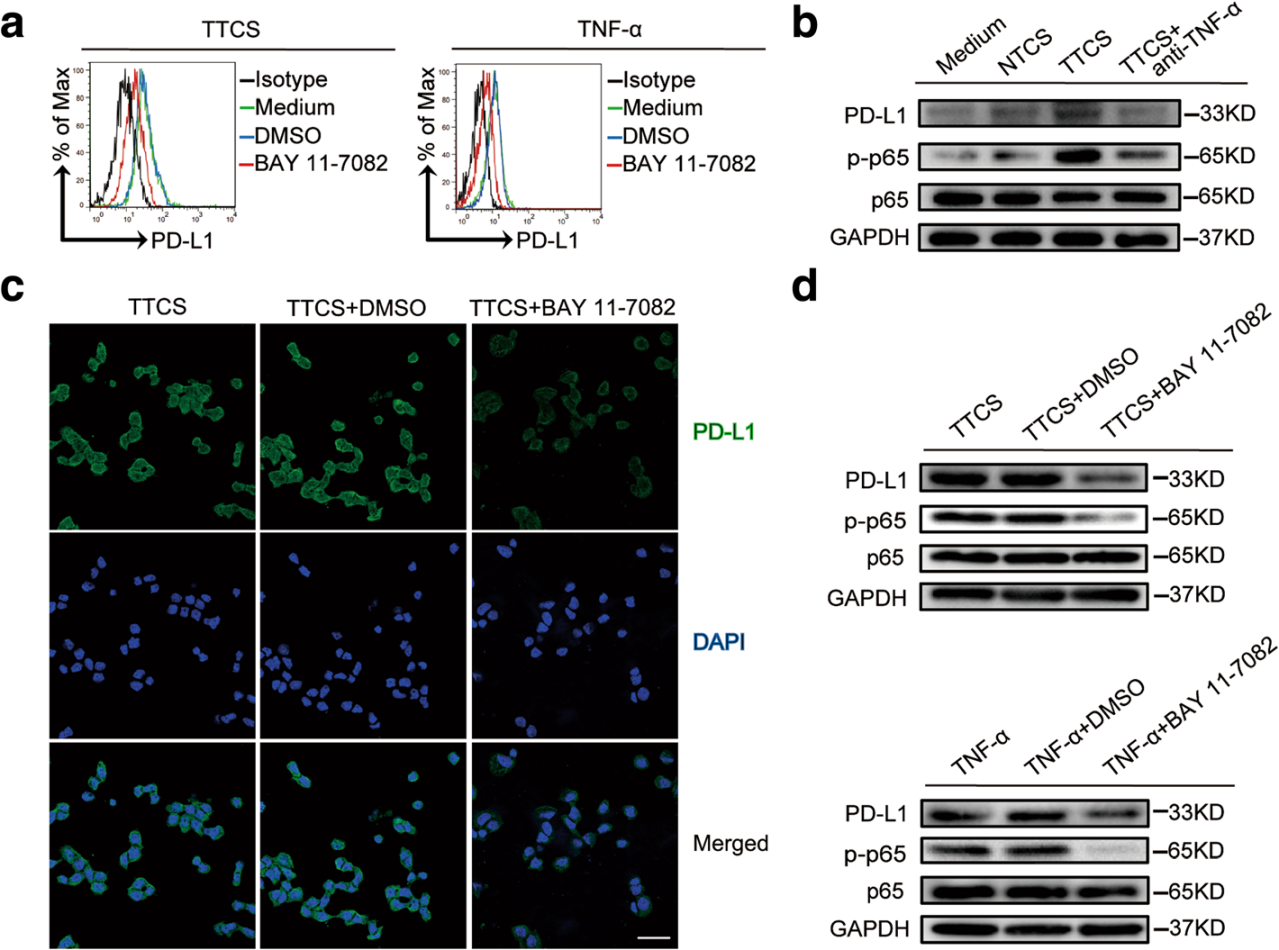
Tumor-derived TNF-α activates NF-κB pathway to induce PD-L1 expression on mast cells. **a** Expression of PD-L1 on hCBMCs exposed to TTCS or TNF-α with or without BAY 11–7082 (an IκBα inhibitor) for 24 h. black, isotype control. **b** PD-L1, p65 and p-p65 on/in LAD2 cells exposed to autologous TTCS, NTCS, or TTCS with anti-TNF-α antibody for 24 h (detecting for PD-L1) or 3 h (detecting for p65 and p-p65) were analyzed by western blot. **c** PD-L1 on HMC-1 cells exposed to TTCS with or without BAY 11–7082 (an IκBα inhibitor) for 24 h was analyzed by immunofluorescence staining. Green, PD-L1; blue, DAPI-stained nuclei. Scale bars: 20 μm. **d** PD-L1, p65 and p-p65 on/in LAD2 cells exposed to TTCS with or without BAY 11–7082 (an IκBα inhibitor) were analyzed by western blot

To further see which signaling pathways might operate in the mast cell PD-L1 induction, we first pre-treated mast cells with corresponding inhibitors and then exposed them to the indicated TTCS. The results showed that only blocking the signal transduction of NF-κB with inhibitor BAY 11–7082 effectively suppressed PD-L1 expression on TTCS-conditioned mast cells or TNF-α-stimulated mast cells (Fig. 4a; Additional file 9: Figure S4a). Furthermore, p65, a direct NF-κB pathway downstream substrate (Fig. 4b), but not other pathway downstream substrates (Additional file 9: Figure S4b), was predominantly more phosphorylated in mast cells after treatment with TTCS than that treatment with NTCS, and this phosphorylation was abolished when blocking TNF-α, implying that activation of NF-κB signaling pathway is crucial for mast cell PD-L1 induction by TNF-α in the GC environments. Similar results were obtained when analyzing PD-L1 on mast cell (Fig. 4b). Furthermore, immunofluorescence staining (Fig. 4c) and western blot (Fig. 4d) showed that PD-L1 expression or p65 phosphorylation was abolished when blocking the signal transduction of NF-κB with inhibitor BAY 11–7082 on mast cells either stimulated with TTCS or TNF-α. Taken together, these data suggest that tumor-derived TNF-α activates NF-κB pathway to induce PD-L1 expression on mast cells.

### Tumor-infiltrating mast cells suppress T cell immunity through PD-L1

**Fig. 5:**
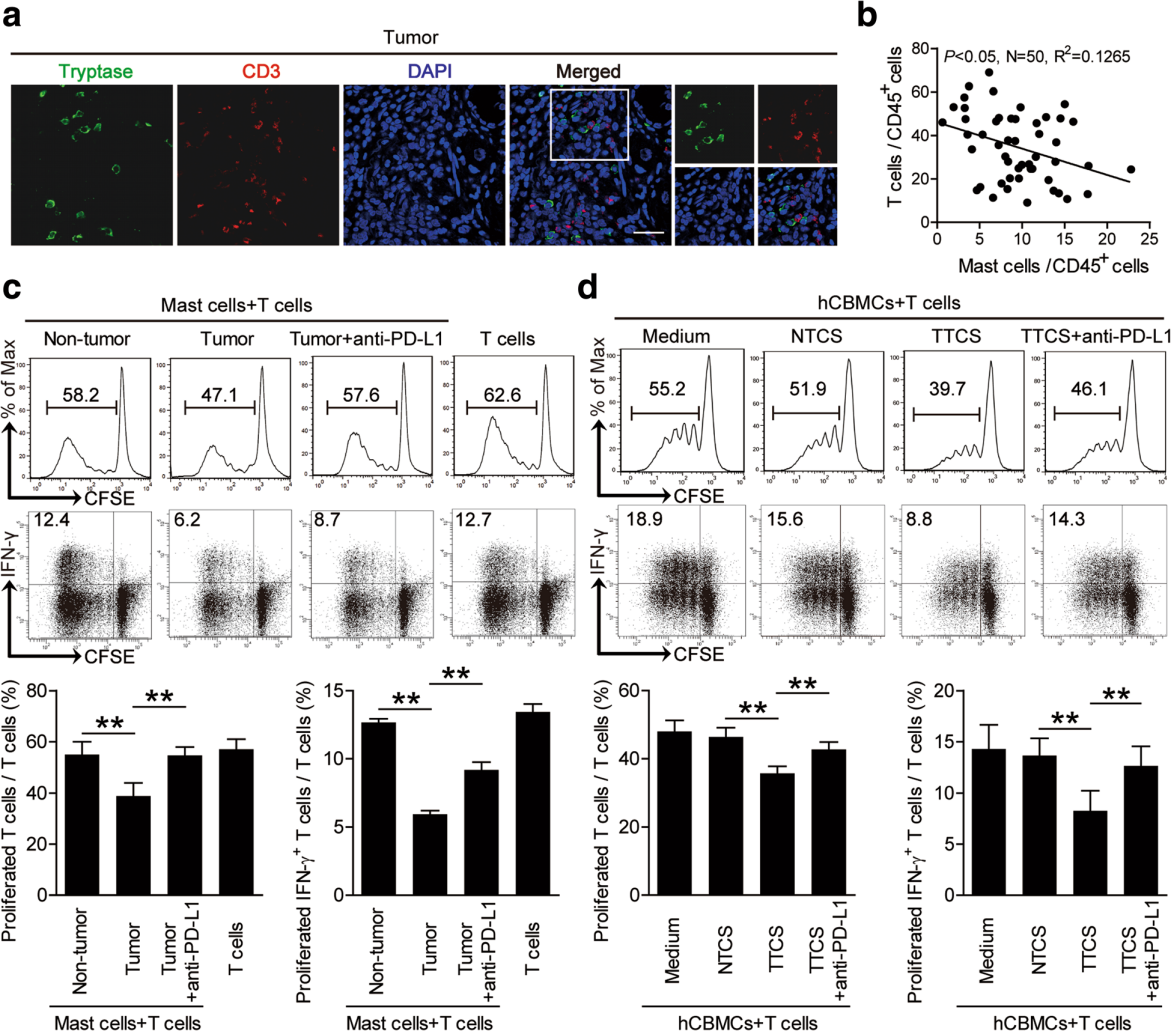
Tumor-infiltrating and tumor-conditioned mast cells suppress T cell immunity through PD-L1. **a** Representative analysis of tryptase^+^ mast cell (green) and CD3^+^ T cell (red) interactions in tumor tissues of GC patients by immunofluorescence. Scale bars: 20 μm. **b** The correlations between mast cells and T cells in human GC tumors were analyzed. Results were expressed as percentage of mast cells and T cells in CD45^+^ cells in tumor tissues of GC patients. **c** CFSE (carboxyfluorescein succinimidyl amino ester)-labeled peripheral CD3^+^ T cells of patients with GC were co-cultured for 5 days with autologous mast cells from non-tumor or tumor tissues with or without anti-PD-L1 antibody. Representative data and statistical analysis of T cell proliferation and IFN-γ production were shown (*n* = 5). **d** CFSE-labeled peripheral CD3^+^ T cells of donors were co-cultured for 5 days with autologous TTCS-, or NTCS-conditioned hCBMCs with or without anti-PD-L1 antibody. Representative data and statistical analysis of T cell proliferation and IFN-γ production were shown (*n* = 5). Each dot in panel **b** represents 1 patient. **, *P* < 0.01 for groups connected by horizontal lines. hCBMCs, human umbilical cord blood-derived cultured mast cells

The co-localization of mast cells and T cells (Fig. 5a) in the tumoral area of GC tissues, and the significant negative correlations between the levels of mast cells and T cells in human GC tumors analyzed (Fig. 5b) suggest that these tumor-infiltrating mast cells may promote tumor progression by impairing T cell immunity. Mast cells from tumor and non-tumor tissues of autologous GC patients were therefore isolated and cultured with purified autologous peripheral CD3^+^ T cells for 5 days. Mast cell/T-cell co-cultures showed that tumor-infiltrating mast cells were superior to non-tumor-derived mast cells in inhibiting T cell proliferation and IFN-γ production (Fig. 5c), suggesting an immunosuppressive function of tumor-infiltrating mast cells in tumor immunity.

Our next objective was to determine the role of PD-L1 on tumor-infiltrating mast cells in T cell suppression. Therefore, we added neutralizing antibodies against PD-L1 into our tumoral mast cell/peripheral T-cell co-culture system. Interestingly, blockade of PD-L1 significantly attenuated such T cell suppression mediated by tumor-infiltrating mast cells (Fig. 5c). Collectively, these findings show that PD-L1 contributes to tumor-infiltrating mast cell-mediated T cell suppression in vitro.

Given the tumor-infiltrating mast cells suppressed T-cell proliferation and IFN-γ production more than non-tumor mast cells, we hypothesized that the tumor microenvironment might have played an important role in this process. To test this, purified peripheral CD3^+^ T cells were co-cultured with TTCS- or NTCS-conditioned primary human umbilical cord blood-derived cultured mast cells (hCBMCs) for 5 days. Consistent with our hypothesis, TTCS-conditioned hCBMCs showed significantly more suppression of T cell proliferation and IFN-γ production (Fig. 5d). To see whether PD-L1 operates in this T cell suppression, we added PD-L1 neutralizing antibodies in the T cell/TTCS-conditioned hCBMCs co-culture system. As expected, PD-L1 blocking by antibody efficiently attenuated such T cell suppression mediated by TTCS-conditioned hCBMCs (Fig. 5d). Similar observations were made when using human mast cell line LAD2 cells (Additional file 10: Figure S5a). Besides, tumor-associated mast cells could modulate cytotoxic function of T cells as perforin and granzyme B production from the T cells cocultured with PD-L1-expressing mast cells decreased significantly (Additional file 10: Figure S5d and e). These results above indicate that, in the GC microenvironment, mast cells acquire ability to suppress T cell function through PD-L1.

### Blockade of mast cell-associated PD-L1 on T cell immunity inhibits tumor growth and GC progression

**Fig. 6:**
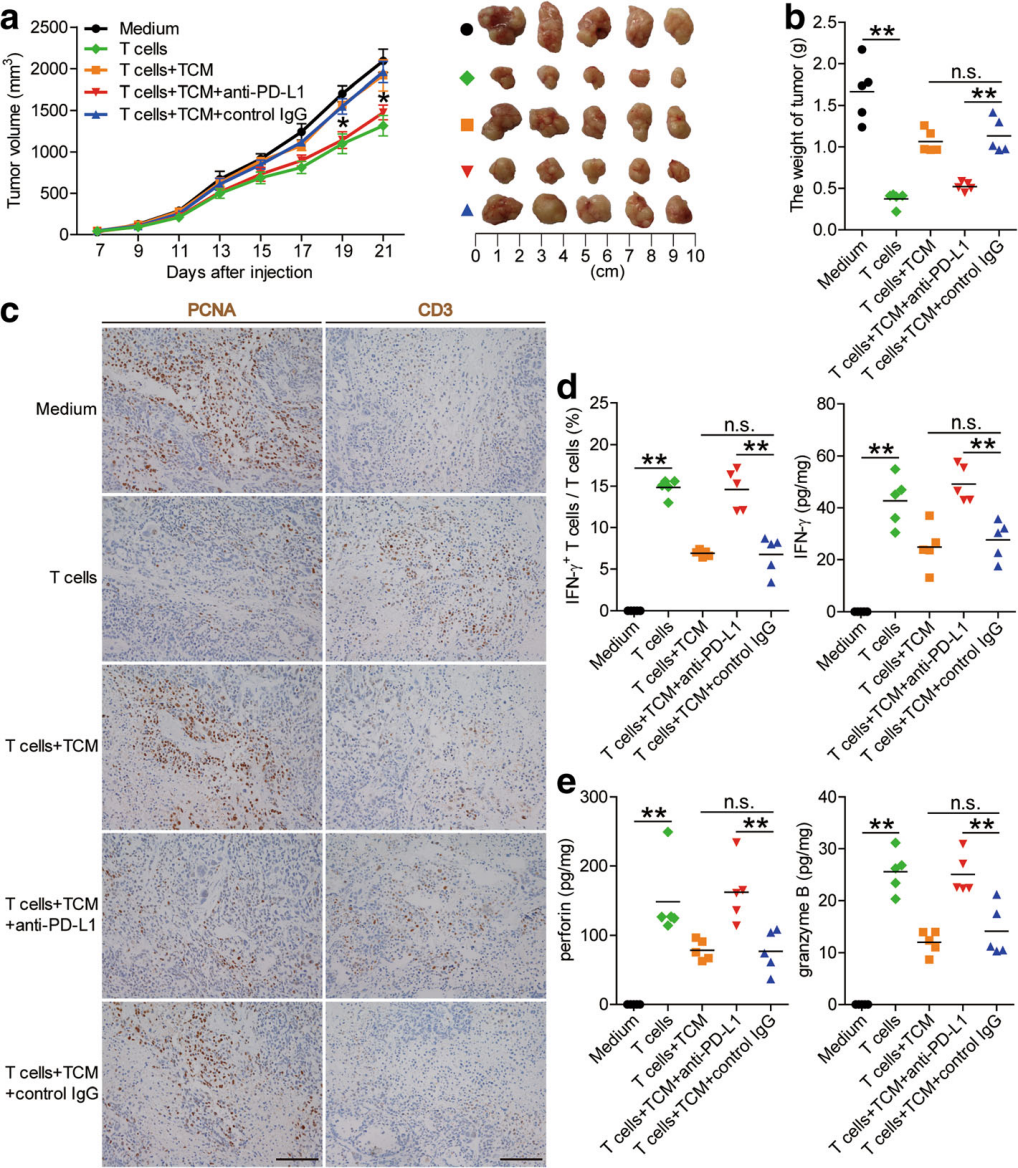
Blockade of mast cell-associated PD-L1 on T cell immunity inhibits tumor growth and GC progression in vivo. **a** Mice were injected with human SGC-7901 cells, as described in Materials and methods. The control animals () received no further injections. The experimental treatments entailed injections with T cells () or T cells in combination with TTCS-conditioned mast cells (TCM) (), or T cells in combination with TCM pre-treated with an anti-PD-L1 antibody () or a control IgG (). The illustrated data represent tumor volumes (5 mice in each group). The day of tumor cell injection was counted as day 0. *P < 0.05, for groups injections with T cells in combination with TCM pre-treated with an anti-PD-L1 antibody (), compared with groups injections with T cells in combination with TCM pre-treated with a control IgG (). The tumors were excised and photographed 21 day after injecting the tumor cells. **b** The weights of tumors were compared. **c**-**e** Proliferating cell nuclear antigen (PCNA) (brown) expression, CD3^+^ T cell infiltration (brown) (**c**), or IFN-γ (**d**), perforin and granzyme B (**e**) production in tumors and IFN-γ-producing T cell response (**d**) in spleens of mice were compared (*n* = 5). Scale bars: 100 μm. The horizontal bars represent mean values. **, *P* < 0.01; n.s., *P* > 0.05 for groups connected by horizontal lines

To test the suppressive effect of PD-L1^+^ mast cells on T cell immunity in vivo, we treated TTCS-conditioned mast cells (TCM) with PD-L1 blocking or control IgG and then injected them together with T cells into our established human NOD/SCID mice bearing SGC-7901-derived GC. As expected, mice without T cell transfusions, mice treated with T cells plus TCM or control IgG-treated TCM, showed tumor growth and disease progression (Fig. 6a and b). Consistent with a vital role in assisting tumors of PD-L1^+^ mast cells in vivo, mice treated with T cells plus PD-L1 blocking antibody-treated TCM showed reduced tumor volumes and disease progression at each measuring time point from day 19 (Fig. 6a and b). Moreover, mice treated with T cells plus PD-L1 blocking antibody-treated TCM, also showed decreased tumor cell proliferation, and increased CD3^+^ T cell infiltration and IFN-γ production (Fig. 6c and d; Additional file 11: Figure S6a), as well as an increased production of cytolytic molecules perforin and granzyme B (Fig. 6e; Additional file 11: Figure S6b and c), compared with the mice treated with T cells plus TCM or control IgG-treated TCM. These findings suggest that tumor-associated mast cells suppress T cell immunity in vivo depending on PD-L1 and thereby contribute to tumor growth and GC progression.

## Discussion

Illuminating the roles of host innate and adaptive immune cells within the tumor milieu is crucial for understanding the development and progression of human tumors [17]. Although previous studies have been delineating the functions of adaptive immune cells in GC [18], the roles of innate immune cells remain less well understood. Mast cells are a group of innate immune cells, which have been reported in GC [14, 19], but the phenotype, functional regulation and clinical relevance of mast cells in human GC microenvironment remain largely unclear. In this study, we have shown that within GC mast cells could play a positive role on promoting tumor progression. We have found that the percentage of mast cells in tumors was significantly increased at advanced stages of GC, with a high percentage of mast cells positively correlating with poor overall survival of GC patients. Furthermore, tumor-infiltrating mast cells colocalized with T cells, and tumor-derived TNF-α could induce immunosuppressive mast cells (PD-L1^+^ mast cells) (Fig. 3g). Although less efficiently than professional antigen-presenting cells (APCs), mast cells can upregulate CD69 expression, proliferation and cytokine production in effector T cells [20, 21] and others have been clearly shown to be able to present antigens to T cells [22]. It is therefore easy to envisage, when they express the immune inhibitory ligand PD-L1 they may inhibit T-cell immunity via PD-L1 as shown in in vitro co-culture as well as in vivo in the absence of PD-L1 neutralizing antibody in this study. These results are similar to those from our previous studies on neutrophils, which are also not considered as professional APCs [23]. The pathological role of mast cells within the tumor microenvironment suggests a novel tumor immune escaping mechanism during GC progression.

In humans, mast cell infiltration in tumors influences disease progression and patient survival [24]. Hence, the analysis of mast cell infiltration in GC is a crucial area of clinical investigation. Our data shed some light on the clinical relevance of mast cells in GC. We observed a significant positive association between the percentage/number of mast cells and advanced clinical features of GC, such as lymphatic invasion, tumor size, and tumor stage (Additional file 2: Figure S1). In addition, we uncovered that an increased frequency/number of intratumoral mast cells predicted a lower rate of disease-free survival, which was oppositely correlated with intratumoral mast cell levels (Additional file 12: Figure S7), suggesting that tumor-infiltrating mast cells may become a helpful clinical prognostic marker in the future.

Regardless of patient outcome, the percentage/number of mast cells was notably increased in tumors compared with non-tumor tissues. This may be a consequence of either enhanced proliferation of intratumoral mast cells or an increased migration of mast cells to tumor tissues. As for the little expression of Ki-67 in mast cells in GC, we exclude the possibility of the enhanced proliferation of intratumoral mast cells and predicted that the tumors possibly induced migration of mast cells by chemotaxis. Recently, mast cells had been reported to be attracted into the tumor microenvironment in a SCF-dependent manner [25]. In our case, we identified a new mechanism for mast cell chemotaxis in GC. In comparison with non-tumor tissue, gastric tumors could secrete more CXCL12, which attracted migration of mast cells. Additionally, some investigations showed that mast cells also expressed chemokine receptors such as CXCR1 and CXCR2 in different cancers [26]. In our study, we found that intratumoral mast cells expressed high level CXCR4 but little CCR2, CCR4, CCR5, CCR7, CXCR1, CXCR2 or CXCR7. Therefore, we are the first to define the vital role of CXCL12-CXCR4 chemotaxis in recruiting mast cells in GC. Certainly, some other cells can also express CXCR4 in GC, such as gastric cancer cells [27], myeloid cells [28], myeloid-derived suppressor cells (MDSCs) [29] and so on. Some studies have shown CXCR4 pathway can promote epithelial-mesenchymal transition in gastric cancer cells [30]. In tumor microenvironment, CXCR4^+^ MDSCs and neutrophils can be recruited by CXCL12 and they impair the anti-tumor functions of CD8^+^ cytotoxic T cells leading to increased tumor metastasis [29, 31].

Mast cells have been identified in tumors recently. However, very little is currently known about the phenotype and function of these tumor-infiltrating mast cells. Immunosuppression has been widely acknowledged as a hallmark of cancer [17]. Interestingly, we screened the expression of immunosuppressive molecules on tumor-infiltrating mast cells, and we found that mast cells within GC expressed high level immunosuppressive molecule PD-L1, suggesting that they may play a role to directly modulate effector function. Crosstalk between PD-L1 and PD-1 is one of the main mechanisms leading to immunosuppression of T cells [32]. In our present study, we uncovered that GC tumor-associated mast cells effectively inhibited T-cell’s immune function in a PD-L1–dependent manner in vitro and in vivo. Furthermore, immunofluorescence staining showed that most tumor–infiltrating mast cells localized in close proximity to CD3^+^ T cells in GC, indicating that immunosuppression could be mediated by the interaction of mast cells and T cells in a PD-L1-dependent manner. We are the first to report that the induced PD-L1 expression on tumor-infiltrating mast cells enabled them to suppress T-cell proliferation and IFN-γ production. Consistent with our findings in GC, blocking PD-L1/ PD-1 pathway could also achieve a good prognosis in testicular germ cell tumors [33]. We previously reported that degranulation of mast cells prompted GC progression [34], which offered additional targets to consider for mast cell directed therapies besides target for mast cell-associated PD-L1. In addition, tumor-associated mast cells can also be activated to secrete sufficient amount of biological molecules to mediate tumor progression [35]. Previous studies found that mast cell released histamine via c-Kit/SCF, which increased cholangiocarcinoma growth, and angiogenesis [36]. Moreover, mast-cell-derived mediators: histamine, CXCL1 and CXCL10 could increase thyroid cancer cell survival and DNA synthesis in vitro [37]. In pancreatic cancer, activated mast cells promote tumor progression by IL-13 and tryptase [38]. Importantly, in vivo GC model, we found TTCS-conditioned mast cells effectively inhibited T cell immunity and promoted GC progression, and such T cell suppression was through PD-L1 on the mast cells as blocking PD-L1 reversed it. Certainly, some other cell types are also known to mediate T-cell suppression in tumor microenvironment. For instance, a recent study showed that LC3-associated phagocytosis (LAP) in myeloid cells regulated macrophages in the tumor microenvironment, which as a result suppressed T cell function and promoted tumor tolerance [39]; and the number of tumor-infiltrating myeloid cells was correlated with early metastatic relapse [40]. Furthermore, it has been recognized that tumors often recruit MDSCs that inhibits T cell infiltration and activation [41]. Therefore tumor resistance to single-agent immune checkpoint inhibitor therapy could be a result of lack infiltrating T cells or too many suppressive immune cells [42, 43]. It will be very important to further explore the role of the suppressive, PD-L1-expressing mast cells reported in this study and their relation with other suppressive immune cell types. Here, our results indicate that the important PD-1-PD-L1 immunosuppression pathway operates in GC via mast cells.

PD-L1 is expressed in a wide range of cell types and tissues and shown to be overexpressed with immune activation, such as inflammation or tumor [23, 44]. Previous studies have shown that PD-L1 up-regulation can be induced mainly by activated CD8^+^ cytotoxic T cells-derived interferon-γ in HCC milieu [45]. Meanwhile, our team identified that tumor-derived GM-CSF effectively induces PD-L1 expression on neutrophils via activation of JAK-STAT3 pathway [23]. Besides, research has revealed that tumor-derived hypoxia inducible factor 1a (HIF-1a) upregulated PD-L1 on MDSCs by binding to a hypoxia-response element (HRE) in the PD-L1 proximal promoter [46]. In this study, we verify TNF-α as a crucial pro-inflammatory factor within GC microenvironment, which derived from GC tumor and effectively induces the expression of PD-L1 on mast cells via activation of NF-κB signaling pathway.

The recent success of checkpoint inhibitor monotherapy opens up a new era of cancer treatment, however, it’s not suited for everyone. How to achieve precision immunotherapy further, select the preponderant beneficiaries, and improve the efficacy of pharmacoeconomics are important topics. Tumor mutational burden (TMB) is a promising new biomarker for predicting the efficacy of cancer immunotherapy, which is measured by whole-exome sequencing and associated with clinical benefit from multiple checkpoint inhibitors. TMB exhibited joint predictive utility in identifying responders and non-responders to the PD-1 antibody pembrolizumab, the number and type of alterations may prove to be valuable for judging the potential usefulness of immune checkpoint inhibitors [47]. Besides, high TMB may be a response biomarker for PD-1/PD-L1 blockade in tumors such as melanoma and non-small cell lung cancer (NSCLC), and results show a linear correlation between higher TMB and favorable outcome parameters with anti-PD-1/PD-L1 monotherapy [48]. In addition, PD-1/PD-L1 also plays an important role in some inflammatory disease. Asthma is a chronic airway inflammation associated largely with CD4^+^ T cells, eosinophils, and mast cells. PD-L1 expressed on mast cells was shown to regulate T-cell activation and tolerance. And PD-L1^+^ mast cell is a negative regulator of conventional CD4^+^ T cells, and understanding of the role will give propulsion to the development of novel therapeutic approaches in allergic asthma [49]. Besides, alopecia areata (AA) is a CD8^+^ T-cell dependent autoimmune disease and mast cells are crucial immunomodulatory cells implicated in the regulation of T cell-dependent immunity in AA. A report showed that a number of PD-L1^+^ mast cells appeared to be further reduced in lesional AA skin compared to healthy skin, particularly during their interactions with CD8^+^ T-cells. Mast cells in AA are skewed towards pro-inflammatory activities and that MC-CD8^+^ T-cell interactions in AA are predominantly pro-inflammatory. This finding suggests that treatment regimens which promote an immune-inhibitory phenotype and/or suppress the switch towards a pro-inflammatory mast cells phenotype, should down-regulate undesired CD8^+^ T-cell responses in AA [50].

## Conclusion

**Fig. 7:**
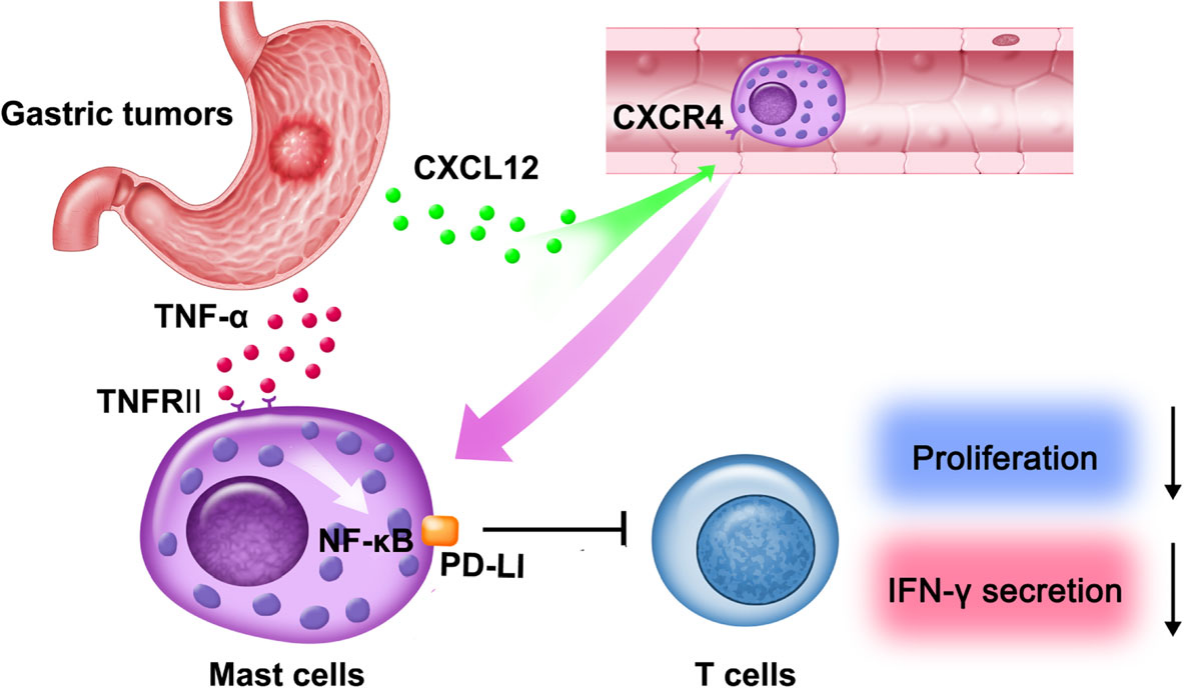
A proposed model of cross-talks among mast cells, T cells, and tumor cells leading to mast cell-mediated immunosuppressive and protumorigenic effects in the GC microenvironment. CXCL12-CXCR4 chemotaxis mediates the recruitment and accumulation of mast cells into the GC microenvironment, which could be up-regulated PD-L1 expression via NF-κB signaling pathway activation by tumor-derived TNF-α. Mast cells inhibit T-cell proliferation and function in a PD-L1-dependent manner in GC, which promotes GC progression

In brief, based on our in vitro and in vivo data, we propose a model involving complex interactions between mast cells, T cells and tumor cells within GC (Fig. 7). First, CXCL12-CXCR4 chemotaxis mediates the recruitment of mast cells into GC microenvironment. And then, tumor-derived TNF-α induces the overexpression of PD-L1 on mast cells via NF-κB signaling pathway activation. Finally, the PD-L1^+^ mast cells inhibit T-cell proliferation and function in a PD-L1-dependent manner. In conclusion, our study has highlighted a notable role for mast cells in human GC and identified new mechanisms of GC-associated mast cells mediating tumor progression. Overall, blocking these pathological mast cells and the TNF-α-PD-L1 immunosuppressive pathway may be a useful therapeutic strategy for preventing GC progress.

## Additional files

Additional file 1 **Table S1.** Antibodies and other reagents. (DOCX 29 kb)

Additional file 2 **Figure S1.** Mast cell percentage (**a**) or mast cell number (**b**) and its potential correlations with clinical parameters. Mast cell percentage in CD45^+^ leukocytes and mast cell number per million total cells were analyzed for correlations with clinical pathological parameters. **, *P* < 0.01; n.s., *P* > 0.05 for groups connected by horizontal lines. Each dot represents one patient. CEA, carcinoembryonic antigen; *H.pylori* Ab, *Helicobacter pylori* antibody. (TIF 1496 kb)

Additional file 3 **Table S3.** Univariate and multivariate analyses of factors associated with survival. (DOCX 20 kb)

Additional file 4 **Table S2.** Clinical characteristics of 114 patients with gastric cancer. (DOCX 19 kb)

Additional file 5 **Table S4.** Correlations between mast cell percentage and clinic pathological features of patients with gastric cancer. (DOCX 20 kb)

Additional file 6 **Table S5.** Correlations between mast cell number and clinic pathological features of patients with gastric cancer. (DOCX 21 kb)

Additional file 7 **Figure S2.** CXCL12-CXCR4 chemotaxis mediates mast cell migration and accumulation in GC tumors. (**a**) Expression of Ki-67 in tumor-infiltrating mast cells by gating on CD45^+^CD117^+^FcεRI^+^ cells. Color histograms represent staining of Ki-67; black, isotype control. (**b**) Tumor-infiltrating tryptase^+^ mast cells and Ki-67^+^ cells were defined by immunofluorescence staining. Green, Tryptase; red, Ki-67; and blue, DAPI-stained nuclei. Scale bars: 50 μm. (**c**) Expression of CCR2, CCR4, CCR5, CCR7, CXCR1, CXCR2 and CXCR7 on tumor-infiltrating mast cells by gating on CD45^+^CD117^+^FcεRI^+^ cells. Color histograms represent staining of chemokine receptors; black, isotype control. (**d**) Representative analysis of CXCL12-expressing (red) EpCam^+^ tumor cells (green) in tumor tissues of GC patients by immunofluorescence. Scale bars: 20 μm. (**e**) Expression of CD80 and CD86 in tumor-infiltrating mast cells by gating on CD45^+^CD117^+^FcεRI^+^ cells. Color histograms represent staining of CD80 and CD86; black, isotype control. (TIF 5879 kb)

Additional file 8 **Figure S3.** Tumor-derived factor TNF-α induces mast cells to express PD-L1. (**a**) Expression of 2B4, glactin-3, CTLA-4, and ICOSL on mast cells by gating on CD45^+^CD117^+^FcεRI^+^ cells. Color histograms represent staining of 2B4, glactin-3, CTLA-4, and ICOSL; black, isotype control. (**b**) Expression of PD-L1 on hCBMCs exposed to IL-1β, IL-6, IL-10, IL-17, IL-22, IL-23, M-CSF, G-CSF, IFN-γ, TGF-β (100 ng/ml) for 24 h. black, isotype control. (**c**) Expression of TNF-α receptor II (TNFRII) on tumor-infiltrating mast cells. Black, isotype control. (TIF 1497 kb)

Additional file 9 **Figure S4.** Tumor-derived TNF-α activates NF-κB pathway to induce PD-L1 expression on mast cells. (**a**) Expression of PD-L1 on hCBMCs exposed to 50% TTCS with or without U0126 (an ERK inhibitor), Wortmannin (a PI3K inhibitor), SB203580 (a MAPK inhibitor), or SP600125 (a JNK inhibitor) for 24 h. black, isotype control. (**b**) p44/42 and p-p44/42, Akt and p-Akt, p38 and p-p38, JNK and p-JNK in LAD2 cells exposed to TTCS with or without anti-TNF-α antibody were analyzed by western blot. (TIF 1181 kb)

Additional file 10 **Figure S5.** Tumor-infiltrating and tumor-conditioned mast cells suppress T cell immunity through PD-L1. (**a**) CFSE-labeled peripheral CD3^+^ T cells of donors were co-cultured for 5 days with TTCS-, or NTCS-conditioned LAD2 cells with or without anti-PD-L1 antibody. Representative data and statistical analysis of T cell proliferation and IFN-γ production were shown (*n* = 5). *, *P* < 0.05; **, *P* < 0.01 for groups connected by horizontal lines. (**b**) Surface staining of FcεRI and CD117 of generated human umbilical cord blood-derived cultured mast cells (hCBMCs) was shown. Results were expressed as percentage of CD117^+^FcεRI^+^ cells by gating on CD45^+^ cells. Iso, Isotype control antibody. (**c**) Toluidine blue staining of sorted human tumor-infiltrating mast cells was shown. Scale bars: 10 μm. (**d**) Granzyme B and perforin production were compared in the supernatants (*n* = 5), which CD3^+^ T cells of patients with GC were co-cultured with autologous mast cells from non-tumor or tumor tissues with or without anti-PD-L1 antibody. (**e**) Granzyme B and perforin production were compared in the supernatants (*n* = 5), which peripheral CD3^+^ T cells of donors were co-cultured with autologous TTCS-, or NTCS-conditioned hCBMCs with or without anti-PD-L1 antibody. **, *P* < 0.01 for groups connected by horizontal lines. hCBMCs, human umbilical cord blood-derived cultured mast cells. (TIF 1676 kb)

Additional file 11 **Figure S6.** Blockade of mast cell-associated PD-L1 on T cell immunity inhibits tumor growth and GC progression in vivo. (**a**-**c**) Mice were injected with human SGC-7901 cells, as described in Materials and methods. The control animals () received no further injections. The experimental treatments entailed injections with T cells () or T cells in combination with TTCS-conditioned mast cells (TCM) (), or T cells in combination with TCM pre-treated with an anti-PD-L1 antibody () or a control IgG (). The illustrated data represent tumor volumes (5 mice in each group). The day of tumor cell injection was counted as day 0. The expression of IFN-γ (**a**), anti-tumor molecules perforin (**b**) and granzyme B (**c**) in tumors of mice on day 21 after tumor cell injection were compared (*n* = 5). The horizontal bars represent mean values. *, *P* < 0.05; **, *P* < 0.01 for groups connected by horizontal lines. (TIF 841 kb)

Additional file 12 **Figure S7.** Kaplan-Meier plots for disease-free survival by median mast cell percentage (9.315%) or median mast cell number (4749 per million). MC (%), mast cell percentage; MC (NO.), mast cell number. (TIF 108 kb)

Additional file 13 **Table S6.** Primer and probe sequences for real-time PCR analysis. (DOCX 20 kb)

Additional file 14 Supplementary Materials and Methods. (DOCX 63 kb)

## Abbreviations

APCs: Antigen-presenting cells
GC: Gastric cancer
hCBMCs: Human umbilical cord blood-derived cultured mast cells
IFN: Interferon
IL: Interleukin
MDSCs: Myeloid-derived suppressor cells
PBMCs: Peripheral blood mononuclear cells
PD-L1: Programmed death-ligand 1
TNF-α: Tumor necrosis factor-α
