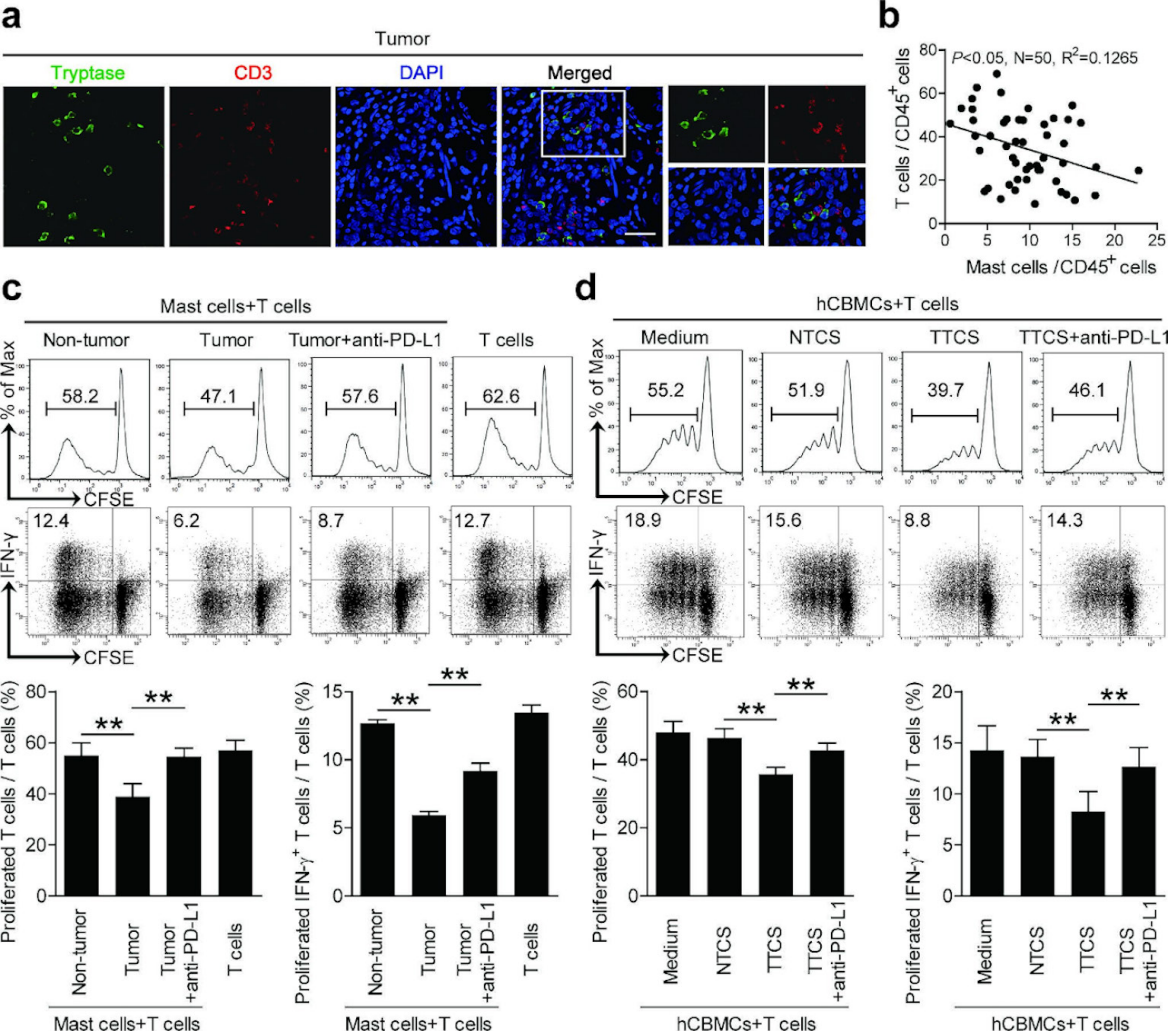# Correction: *Increased intratumoral mast cells foster immune suppression and gastric cancer progression through TNF-α-PD-L1 pathway*

**DOI:** 10.1136/jitc-2020-0530-3corr1

**Published:** 2020-07-28

Lv Y, Zhao Y, Wang X, *et al.* Increased intratumoral mast cells foster immune suppression and gastric cancer progression through TNF-α-PD-L1 pathway. *J ImmunoTher Cancer* 2019;7:54. doi: 10.1186/s40425-019-0530-3

Since the online publication of this article, the authors noticed that the results of tumor-infiltrating mast cells in inhibiting IFN-γ production was mistakenly duplicated in figure 5. The correct is figure is below: