# Supplementary material for: Increased intratumoral mast cells foster immune suppression and gastric cancer progression through TNF-α-PD-L1 pathway
Source: J Immunother Cancer. 2019 Feb 26;7:54. doi: 10.1186/s40425-019-0530-3 (PMC6390584; doi:10.1186/s40425-019-0530-3)
Supplement: Supplementary file 1 — Table S1. Antibodies and other reagents. (DOCX 29 kb) [file 40425_2019_530_MOESM1_ESM.docx]

**Supplementary Table 1.** Antibodies and other reagents

| Antibodies and reagents | Manufacturers |  |
| --- | --- | --- |
| Antibodies for flow cytometry |  |  |
| anti-CD45-PE-Cy7 | Biolegend |  |
| anti-CD117-PerCP-Cy5.5 | Biolegend |  |
| anti-FcεRI-FITC | Biolegend |  |
| anti-Ki-67-PE | Biolegend |  |
| anti-PD-L1-PE  anti-CXCR4-APC  anti-CCR2-APC  anti-CCR4-APC | Biolegend  Biolegend  Biolegend  Biolegend |  |
| anti-CCR5-APC | Biolegend |  |
| anti-CCR7-PE | Biolegend |  |
| anti-CXCR1-APC | Biolegend |  |
| anti-CXCR2-PE | Biolegend |  |
| anti-CXCR7-APC | Biolegend |  |
| anti-2B4-APC | Biolegend |  |
| anti-Glactin-3-APC | Biolegend |  |
| anti-CTLA-4-PE | Biolegend |  |
| anti-ICOSL-PE  anti-CD120b-PE | Biolegend  Biolegend |  |
| anti-CD3-APC-H7 | BD Pharmingen |  |
| anti-IFN-γ-PE-Cy7 | BD Pharmingen |  |
| Antibodies for immunohistochemical staining |  |  |
| anti-human tryptase | Abcam |  |
| Anti-human CD3 | Abcam |  |
| anti-human proliferating cell nuclear antigen (PCNA) | Santa Cruz |  |
| horseradish peroxidase anti-rabbit IgG  horseradish peroxidase anti-mouse IgG  DAB kit | Zhongshan Biotechnology  Zhongshan Biotechnology  Zhongshan Biotechnology |  |
| Antibodies for immunofluorescence  rabbit anti-human CD3  mouse anti-human tryptase  mouse anti-human EpCam  mouse anti-human PD-L1  rabbit anti-human CXCL12  rabbit anti-human CXCR4  rabbit anti-human Ki-67  goat anti-rabbit-TRITC  goat anti-mouse-FITC | Abcam  Abcam  Abcam  Abcam  Santa Cruz  Abcam  Abcam  Zhongshan Biotechnology  Zhongshan Biotechnology |  |
| Antibodies for neutralizing and blocking |  |  |
| anti-human CXCL12 (Mouse IgG1)  Mouse IgG1 Isotype Control | R&D Systems  R&D Systems |  |
| anti-human CXCR4 (Mouse IgG2b)  Mouse IgG2b Isotype Control | R&D Systems  R&D Systems |  |
| anti-human PD-L1  Goat IgG Isotype Control | R&D Systems  R&D Systems |  |
| Antibodies for western blot |  |  |
| anti-human PD-L1  anti-human p-p65  anti-human p65  anti-human p-p44/42  anti-human p44/42  anti-human p-p38  anti-human p38  anti-human p-JNK  anti-human JNK | Abcam  Cell signaling technology  Cell signaling technology  Cell signaling technology  Cell signaling technology  Cell signaling technology  Cell signaling technology  Cell signaling technology  Cell signaling technology | |
| anti-human AKT | Cell signaling technology |  |
| anti-human p-AKT  anti-human GAPDH | Cell signaling technology  Beijing Ray Antibody Biotech |  |
| Purified anti-CD3 and anti-CD28 antibodies | Biolegend |  |
| ELISA kits |  |  |
| TNF-α  CXCL12 | Biolegend  R&D Systems | |
| IFN-γ | Biolegend |  |
| Perforin  Granzyme B | Abcam  Invitrogen |  |
| Reagents for signaling pathways inhibition |  |  |
| MEK-1 and MEK-2 inhibitor U0126 | Merk Millipore |  |
| IκBα inhibitor BAY 11-7082 | Calbiochem |  |
| JNK inhibitor SP600125 | Calbiochem |  |
| MAPK inhibitor SB203580 | Calbiochem |  |
| PI3K inhibitor Wortmannin | Calbiochem |  |
| CD133 microbeads | Milteniy Biotec |  |
| CD3 microbeads | Milteniy Biotec |  |
| 8-μm pore size Transwells | Corning |  |
| glycine | Sangon Biotech |  |
| Collagenase IV | Gibco |  |
| DNase I | Sigma-Aldrich |  |
| Phorbol myristate acetate | Sigma-Aldrich |  |
| Ionomycin | Sigma-Aldrich |  |
| DMSO | Sigma-Aldrich |  |
| Golgistop and Perm/Wash solution | BD Pharmingen |  |
| Carboxylfluorescein succinimidyl ester (CFSE) | eBioscience |  |
| Protein Extraction Reagent | Pierce |  |
| SuperSignal® West Dura Extended Duration Substrate kit | Thermo |  |
| Fetal calf serum (FCS) | Gibco |  |
| Penicillin/Streptomycin | Gibco |  |
| RPMI-1640 | Hyclone |  |
| StemSpan Serum-Free Expansion Medium  Serum-Free Media (StemPro-34) | StemCell Technologies  Life Technologies |  |
| L-glutamine | Gibco |  |
| Ficoll-Paque Plus | GE Healthcare |  |
| lyses solution  TRIzol reagent | TIANGEN  Invitrogen |  |
| PrimeScriptTM RT reagent Kit | TaKaRa |  |
| Real-time PCR Master Mix | Toyobo |  |
| All recombinant human/mouse cytokines and chemokines | PeproTech |  |

APC-Cy7, allophycocyanin-cyanin 7; PE-Cy7, phycoerythrin-cyanin 7; FITC, Fluorescein isothiocyanate; PE, phycoerythrin; PerCP-Cy5.5, peridin chlorophyl protein-cyanin 5.5; APC, allophycocyanin; IL, interleukin; IFN, interferon; TNF, Tumor Necrosis Factor; PD-L1, programmed death-ligand 1; EpCam, epithelial cell adhesion molecule; PCNA, proliferating cell nuclear antigen.
