# Supplementary figures and images for: Increased intratumoral mast cells foster immune suppression and gastric cancer progression through TNF-α-PD-L1 pathway
Source: J Immunother Cancer. 2019 Feb 26;7:54. doi: 10.1186/s40425-019-0530-3 (PMC6390584; doi:10.1186/s40425-019-0530-3)

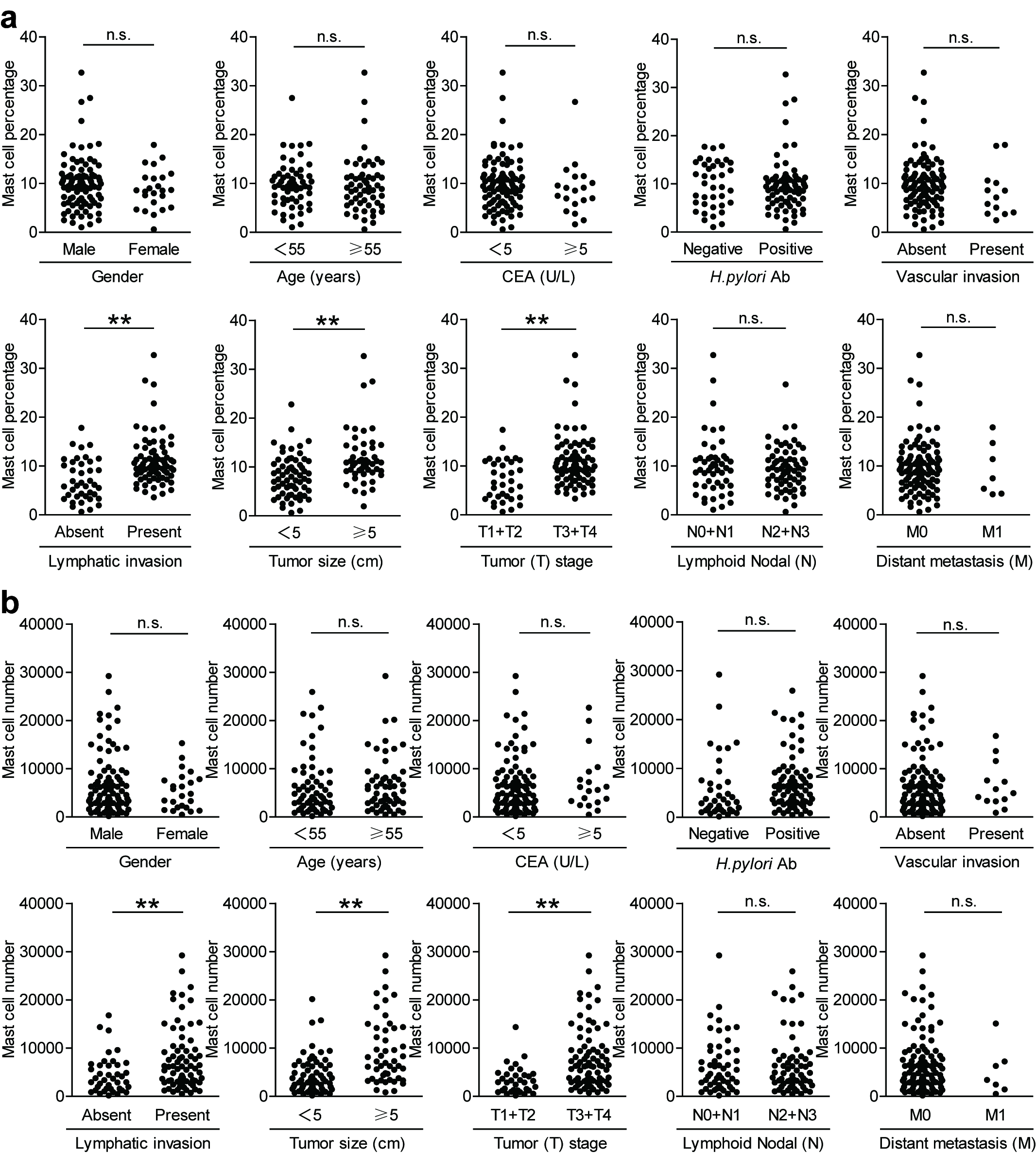

Supplement: Supplementary file 2 — Figure S1. Mast cell percentage (a) or mast cell number (b) and its potential correlations with clinical parameters. Mast cell percentage in CD45+ leukocytes and mast cell number per million total cells were analyzed for correlations with clinical pathological parameters. **, P < 0.01; n.s., P > 0.05 for groups connected by horizontal lines. Each dot represents one patient. CEA, carcinoembryonic antigen; H.pylori Ab, Helicobacter pylori antibody. (TIF 1496 kb) [file 40425_2019_530_MOESM2_ESM.tif]

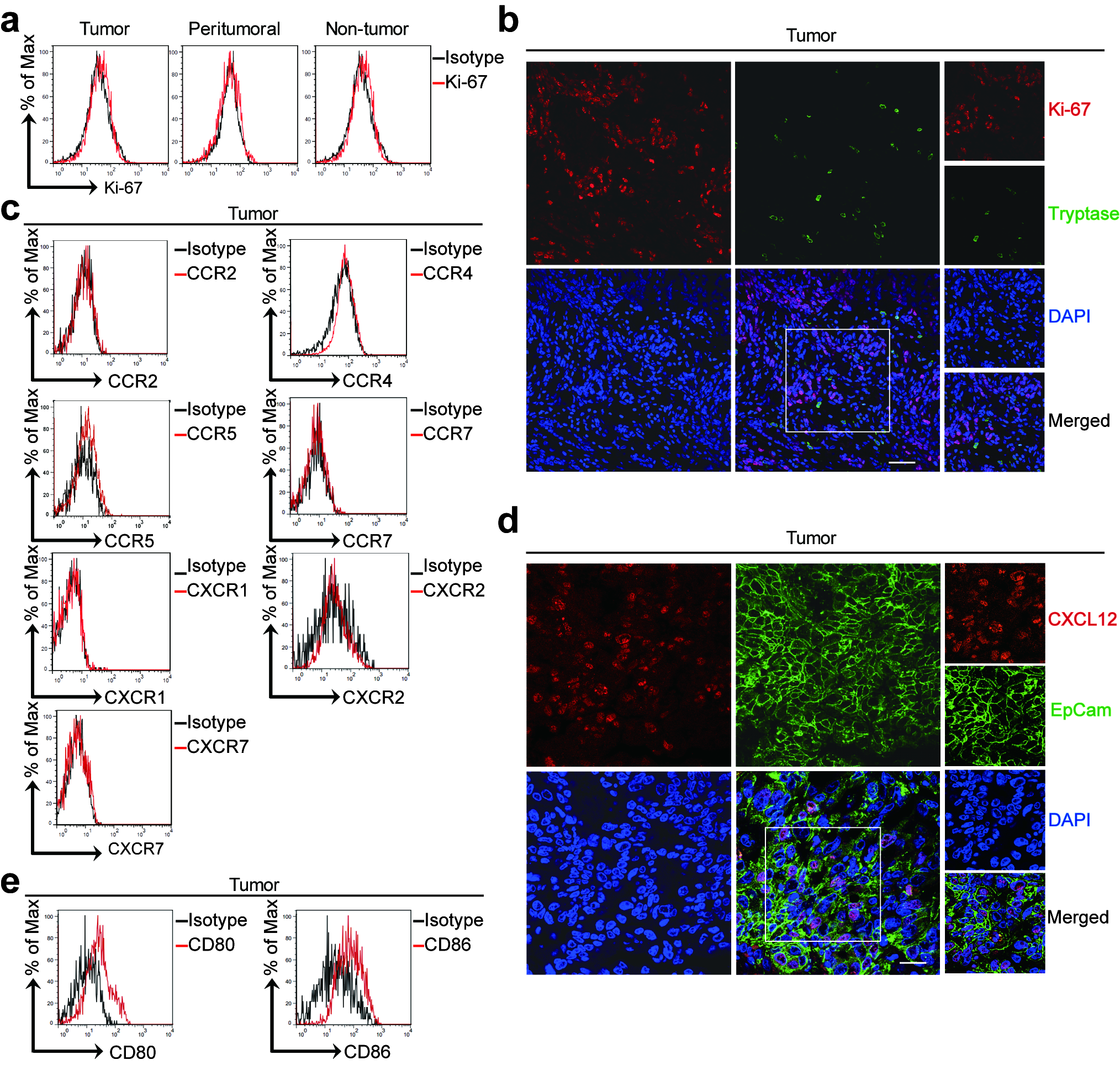

Supplement: Supplementary file 7 — Figure S2. CXCL12-CXCR4 chemotaxis mediates mast cell migration and accumulation in GC tumors. (a) Expression of Ki-67 in tumor-infiltrating mast cells by gating on CD45+CD117+FcεRI+ cells. Color histograms represent staining of Ki-67; black, isotype control. (b) Tumor-infiltrating tryptase+ mast cells and Ki-67+ cells were defined by immunofluorescence staining. Green, Tryptase; red, Ki-67; and blue, DAPI-stained nuclei. Scale bars: 50 μm. (c) Expression of CCR2, CCR4, CCR5, CCR7, CXCR1, CXCR2 and CXCR7 on tumor-infiltrating mast cells by gating on CD45+CD117+FcεRI+ cells. Color histograms represent staining of chemokine receptors; black, isotype control. (d) Representative analysis of CXCL12-expressing (red) EpCam+ tumor cells (green) in tumor tissues of GC patients by immunofluorescence. Scale bars: 20 μm. (e) Expression of CD80 and CD86 in tumor-infiltrating mast cells by gating on CD45+CD117+FcεRI+ cells. Color histograms represent staining of CD80 and CD86; black, isotype control. (TIF 5879 kb) [file 40425_2019_530_MOESM7_ESM.tif]

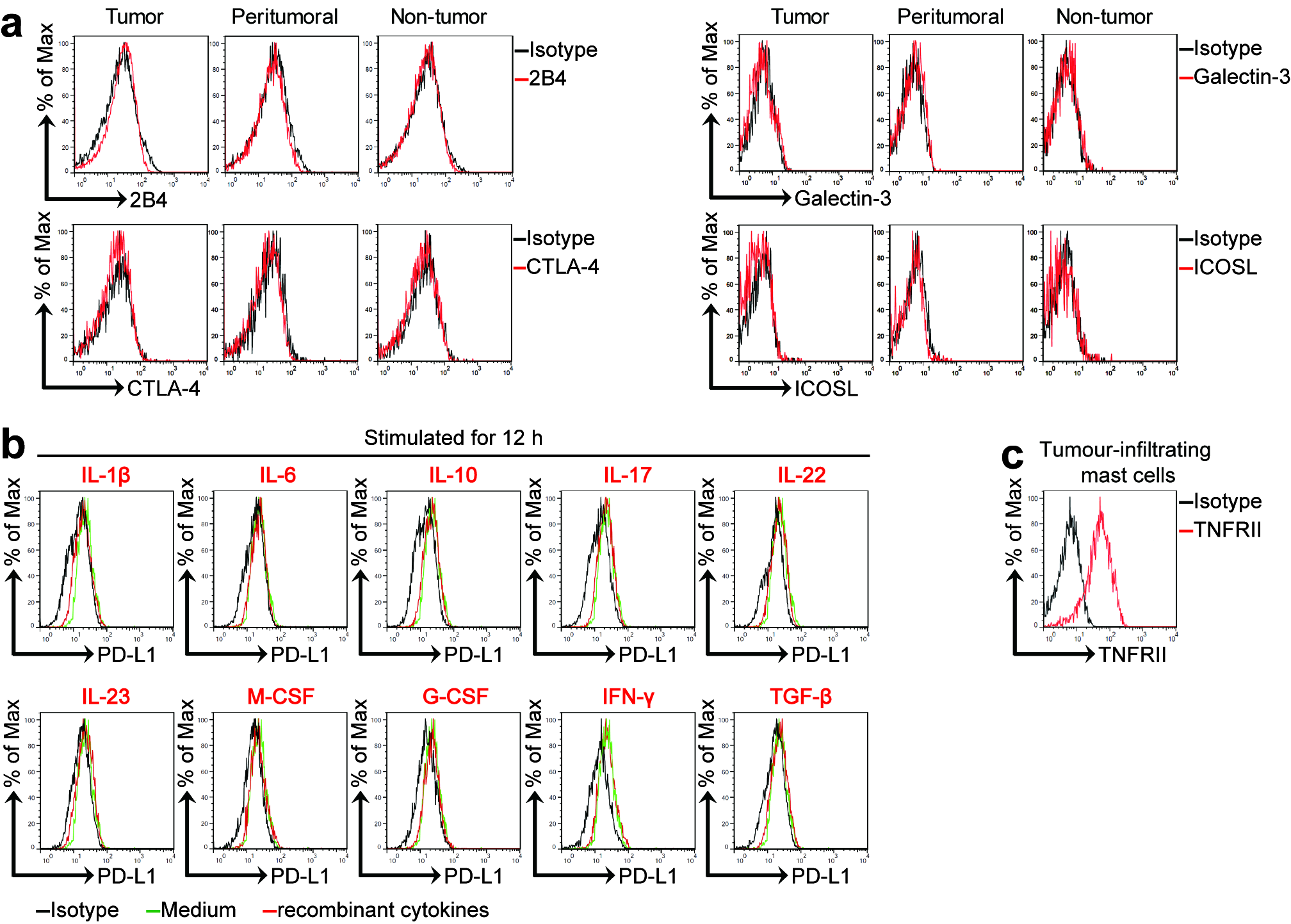

Supplement: Supplementary file 8 — Figure S3. Tumor-derived factor TNF-α induces mast cells to express PD-L1. (a) Expression of 2B4, glactin-3, CTLA-4, and ICOSL on mast cells by gating on CD45+CD117+FcεRI+ cells. Color histograms represent staining of 2B4, glactin-3, CTLA-4, and ICOSL; black, isotype control. (b) Expression of PD-L1 on hCBMCs exposed to IL-1β, IL-6, IL-10, IL-17, IL-22, IL-23, M-CSF, G-CSF, IFN-γ, TGF-β (100 ng/ml) for 24 h. black, isotype control. (c) Expression of TNF-α receptor II (TNFRII) on tumor-infiltrating mast cells. Black, isotype control. (TIF 1497 kb) [file 40425_2019_530_MOESM8_ESM.tif]

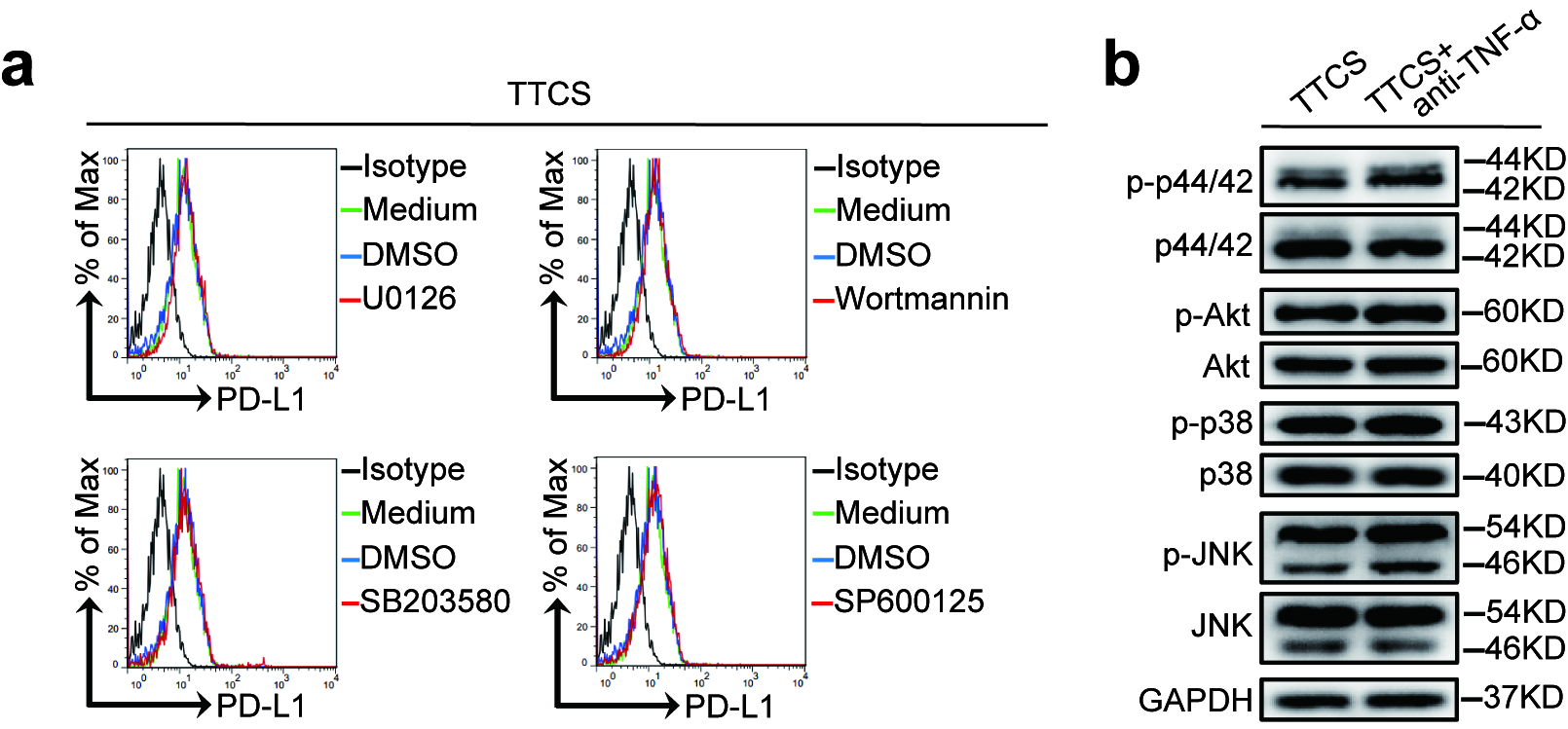

Supplement: Supplementary file 9 — Figure S4. Tumor-derived TNF-α activates NF-κB pathway to induce PD-L1 expression on mast cells. (a) Expression of PD-L1 on hCBMCs exposed to 50% TTCS with or without U0126 (an ERK inhibitor), Wortmannin (a PI3K inhibitor), SB203580 (a MAPK inhibitor), or SP600125 (a JNK inhibitor) for 24 h. black, isotype control. (b) p44/42 and p-p44/42, Akt and p-Akt, p38 and p-p38, JNK and p-JNK in LAD2 cells exposed to TTCS with or without anti-TNF-α antibody were analyzed by western blot. (TIF 1181 kb) [file 40425_2019_530_MOESM9_ESM.tif]

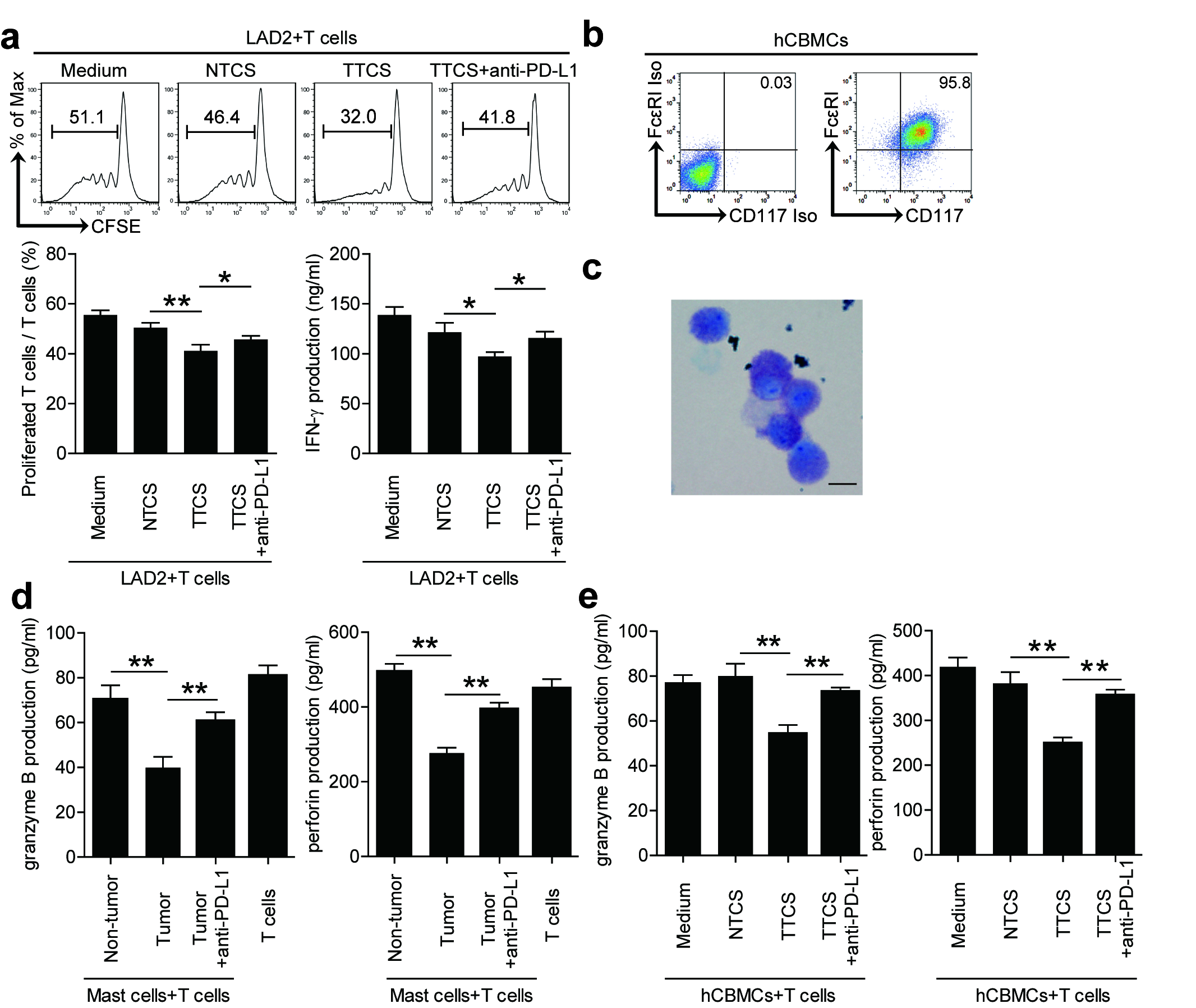

Supplement: Supplementary file 10 — Figure S5. Tumor-infiltrating and tumor-conditioned mast cells suppress T cell immunity through PD-L1. (a) CFSE-labeled peripheral CD3+ T cells of donors were co-cultured for 5 days with TTCS-, or NTCS-conditioned LAD2 cells with or without anti-PD-L1 antibody. Representative data and statistical analysis of T cell proliferation and IFN-γ production were shown (n = 5). *, P < 0.05; **, P < 0.01 for groups connected by horizontal lines. (b) Surface staining of FcεRI and CD117 of generated human umbilical cord blood-derived cultured mast cells (hCBMCs) was shown. Results were expressed as percentage of CD117+FcεRI+ cells by gating on CD45+ cells. Iso, Isotype control antibody. (c) Toluidine blue staining of sorted human tumor-infiltrating mast cells was shown. Scale bars: 10 μm. (d) Granzyme B and perforin production were compared in the supernatants (n = 5), which CD3+ T cells of patients with GC were co-cultured with autologous mast cells from non-tumor or tumor tissues with or without anti-PD-L1 antibody. (e) Granzyme B and perforin production were compared in the supernatants (n = 5), which peripheral CD3+ T cells of donors were co-cultured with autologous TTCS-, or NTCS-conditioned hCBMCs with or without anti-PD-L1 antibody. **, P < 0.01 for groups connected by horizontal lines. hCBMCs, human umbilical cord blood-derived cultured mast cells. (TIF 1676 kb) [file 40425_2019_530_MOESM10_ESM.tif]

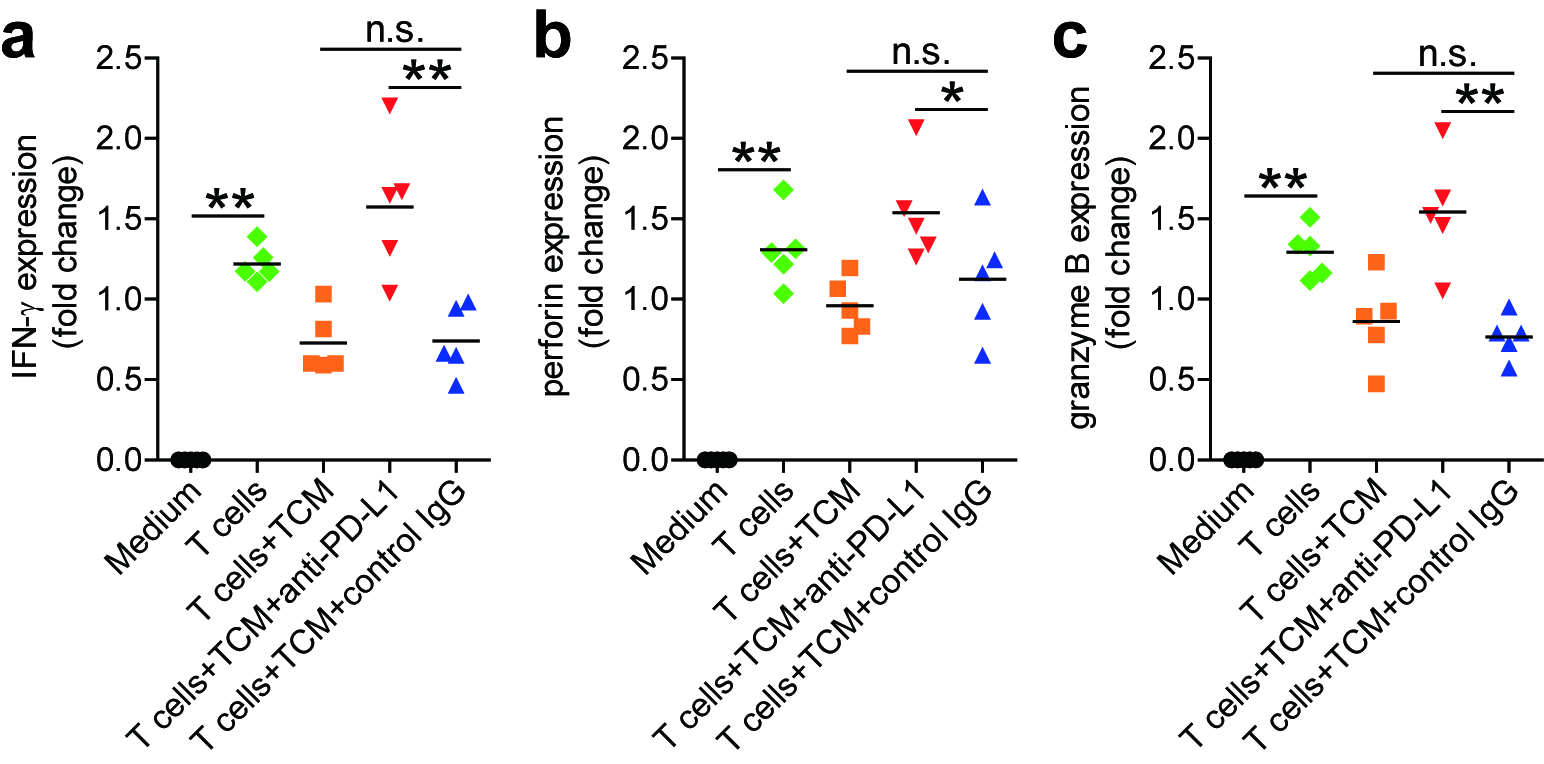

Supplement: Supplementary file 11 — Figure S6. Blockade of mast cell-associated PD-L1 on T cell immunity inhibits tumor growth and GC progression in vivo. (a-c) Mice were injected with human SGC-7901 cells, as described in Materials and methods. The control animals () received no further injections. The experimental treatments entailed injections with T cells () or T cells in combination with TTCS-conditioned mast cells (TCM) (), or T cells in combination with TCM pre-treated with an anti-PD-L1 antibody () or a control IgG (). The illustrated data represent tumor volumes (5 mice in each group). The day of tumor cell injection was counted as day 0. The expression of IFN-γ (a), anti-tumor molecules perforin (b) and granzyme B (c) in tumors of mice on day 21 after tumor cell injection were compared (n = 5). The horizontal bars represent mean values. *, P < 0.05; **, P < 0.01 for groups connected by horizontal lines. (TIF 841 kb) [file 40425_2019_530_MOESM11_ESM.tif]

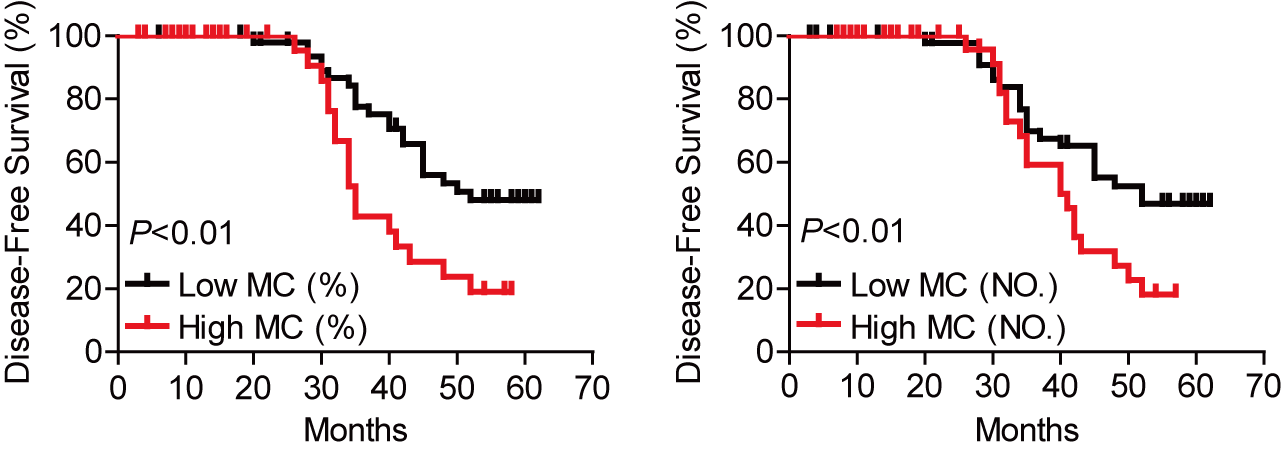

Supplement: Supplementary file 12 — Figure S7. Kaplan-Meier plots for disease-free survival by median mast cell percentage (9.315%) or median mast cell number (4749 per million). MC (%), mast cell percentage; MC (NO.), mast cell number. (TIF 108 kb) [file 40425_2019_530_MOESM12_ESM.tif]
