# Supplementary material for: Increased intratumoral mast cells foster immune suppression and gastric cancer progression through TNF-α-PD-L1 pathway
Source: J Immunother Cancer. 2019 Feb 26;7:54. doi: 10.1186/s40425-019-0530-3 (PMC6390584; doi:10.1186/s40425-019-0530-3)
Supplement: Supplementary file 3 — Table S3. Univariate and multivariate analyses of factors associated with survival. (DOCX 20 kb) [file 40425_2019_530_MOESM3_ESM.docx]

**Supplementary Table 3.** Univariate and multivariate analyses of factors associated with survival

| Variables | Univariate | Multivariate | | |
| --- | --- | --- | --- | --- |
|  | *P*-value | HR | 95% CI | *P*-value |
| Gender (male vs. female) | 0.221 |  |  | NA |
| Age, years (≥ 55 vs. < 55) | 0.967 |  |  | NA |
| *H.pylori* Ab (positive vs. negative) | 0.051 |  |  | NA |
| CEA,U/L (≥ 5 vs. < 5) | 0.106 |  |  | NA |
| Tumor size, cm (≥ 5 vs. < 5) | 0.708 |  |  | NA |
| Lymphatic invasion (positive vs. negative) | 0.162 |  |  | NA |
| Vascular invasion (positive vs. negtive) | 0.140 |  |  | NA |
| Tumor (T) invasion (T1+T2 vs. T3+T4) | 0.043 | 0.557 | 0.310-1.001 | 0.050 |
| Lymphoid Nodal (N) status (N0+N1 vs. N2+N3) | 0.899 |  |  | NA |
| Distant metastasis (M) status (M0 vs. M1) | 0.254 |  |  | NA |
| TNM stage (I+II/III+IV) | 0.620 |  |  | NA |
| Mast cell percentage^a^ (high vs. low) | 0.026 | 2.160 | 1.049-4.448 | 0.037 |
| Mast cell number^b^ (high vs. low) | 0.058 | 2.036 | 1.129-3.671 | 0.018 |

Cox proportional hazards regression model. Variables used in multivariate analysis were adopted by univariate analysis. ^a^Mast cell percentage was acquired on CD117^+^FcεRI^+^ cells that gated on CD45^+^ leukocytes of tumor tissues. ^b^Mast cell number was acquired by counting CD45^+^CD117^+^FcεRI^+^ cells per million cells of tumor tissues. CEA, carcinoembryonic antigen; *H.pylori* Ab, *Helicobacter pylori* antibody; HR, hazard ratio; CI, confidence interval; NA, not adopted.
