# Supplementary material for: Increased intratumoral mast cells foster immune suppression and gastric cancer progression through TNF-α-PD-L1 pathway
Source: J Immunother Cancer. 2019 Feb 26;7:54. doi: 10.1186/s40425-019-0530-3 (PMC6390584; doi:10.1186/s40425-019-0530-3)
Supplement: Supplementary file 4 — Table S2. Clinical characteristics of 114 patients with gastric cancer. (DOCX 19 kb) [file 40425_2019_530_MOESM4_ESM.docx]

**Supplementary Table 2.** Clinical characteristics of 114 patients with gastric cancer

| Variables | No. of patients |
| --- | --- |
| Gender (male/female) | 90/24 |
| Age (years; median, range) | 57, 25-82 |
| *H.pylori* Ab (negative/positive) | 38/76 |
| CEA (U/L; <5/≥5) | 95/19 |
| Tumor size (cm; <5/≥5) | 65/49 |
| Lymphatic invasion (absent/present) | 40/74 |
| Vascular invasion (absent/present) | 101/13 |
| Tumor (T) invasion (T1+T2/T3+T4) | 31/83 |
| Lymphoid Nodal (N) status (N0+N1/N2+N3) | 50/64 |
| Distant metastasis (M) status (M0/M1) | 107/7 |
| TNM stage (I+II/III+IV) | 48/66 |
| Mast cell percentage^a^ (median, range) | 9.315, 0.626-32.7 |
| Mast cell number^b^ (median, range) | 4749, 183-29236 |

^a^Mast cell percentage was acquired on CD117^+^FcεRI^+^ cells that gated on CD45^+^ leukocytes of tumor tissues. ^b^Mast cell number was acquired by counting CD45^+^CD117^+^FcεRI^+^ cells per million cells of tumor tissues. CEA, carcinoembryonic antigen; *H.pylori* Ab, *Helicobacter pylori* antibody.
