# Supplementary material for: Increased intratumoral mast cells foster immune suppression and gastric cancer progression through TNF-α-PD-L1 pathway
Source: J Immunother Cancer. 2019 Feb 26;7:54. doi: 10.1186/s40425-019-0530-3 (PMC6390584; doi:10.1186/s40425-019-0530-3)
Supplement: Supplementary file 5 — Table S4. Correlations between mast cell percentage and clinic pathological features of patients with gastric cancer. (DOCX 20 kb) [file 40425_2019_530_MOESM5_ESM.docx]

**Supplementary Table 4.** Correlations between mast cell percentage and clinic pathological features of patients with gastric cancer

| Variables | Mast cell percentage^a^ | | *P*-value |
| --- | --- | --- | --- |
|  | Low | High |  |
| Gender |  |  |  |
| Male | 42 | 48 |  |
| Female | 15 | 9 | 0.168 |
| Age (years) |  |  |  |
| < 55 | 27 | 31 |  |
| ≥ 55 | 30 | 26 | 0.454 |
| *H.pylori* Ab |  |  |  |
| Negative | 17 | 21 |  |
| Positive | 39 | 37 | 0.508 |
| CEA (U/L) |  |  |  |
| < 5 | 45 | 50 |  |
| ≥ 5 | 12 | 7 | 0.209 |
| Tumor size (cm) |  |  |  |
| < 5 | 42 | 23 |  |
| ≥ 5 | 15 | 34 | <0.001 |
| Lymphatic invasion |  |  |  |
| Absent | 28 | 12 |  |
| Present | 29 | 45 | 0.002 |
| Vascular invasion |  |  |  |
| Absent | 46 | 55 |  |
| Present | 9 | 4 | 0.108 |
| Tumor (T) invasion |  |  |  |
| T1+T2 | 23 | 11 |  |
| T3+T4 | 34 | 46 | 0.014 |
| Lymphoid Nodal (N) status |  |  |  |
| N0+N1 | 25 | 24 |  |
| N2+N3 | 32 | 33 | 0.850 |
| Distant metastasis (M) status |  |  |  |
| M0 | 52 | 55 |  |
| M1 | 4 | 3 | 0.661 |
| TNM stage |  |  |  |
| I+II | 32 | 16 |  |
| III+IV | 22 | 44 | <0.001 |

^a^Mast cell percentage was acquired on CD117^+^FcεRI^+^ cells in CD45^+^ leukocytes that gated on CD45^+^ leukocytes of tumor tissues. CEA, carcinoembryonic antigen; *H.pylori* Ab, *Helicobacter pylori* antibody.
