# Supplementary material for: Increased intratumoral mast cells foster immune suppression and gastric cancer progression through TNF-α-PD-L1 pathway
Source: J Immunother Cancer. 2019 Feb 26;7:54. doi: 10.1186/s40425-019-0530-3 (PMC6390584; doi:10.1186/s40425-019-0530-3)
Supplement: Supplementary file 6 — Table S5. Correlations between mast cell number and clinic pathological features of patients with gastric cancer. (DOCX 21 kb) [file 40425_2019_530_MOESM6_ESM.docx]

**Supplementary Table 5.** Correlations between mast cell number and clinic pathological features of patients with gastric cancer

| Variables | Mast cell number^a^ | | *P*-value |
| --- | --- | --- | --- |
|  | Low | High |  |
| Gender |  |  |  |
| Male | 45 | 45 |  |
| Female | 12 | 12 | 1.000 |
| Age (years) |  |  |  |
| < 55 | 31 | 28 |  |
| ≥ 55 | 26 | 29 | 0.574 |
| *H.pylori* Ab |  |  |  |
| Negative | 14 | 24 |  |
| Positive | 33 | 43 | 0.501 |
| CEA (U/L) |  |  |  |
| < 5 | 49 | 46 |  |
| ≥ 5 | 8 | 11 | 0.451 |
| Tumor size (cm) |  |  |  |
| < 5 | 43 | 22 |  |
| ≥ 5 | 15 | 34 | <0.001 |
| Lymphatic invasion |  |  |  |
| Absent | 26 | 14 |  |
| Present | 31 | 43 | 0.019 |
| Vascular invasion |  |  |  |
| Absent | 51 | 50 |  |
| Present | 6 | 7 | 0.768 |
| Tumor (T) invasion |  |  |  |
| T1+T2 | 27 | 7 |  |
| T3+T4 | 30 | 50 | <0.001 |
| Lymphoid Nodal (N) status |  |  |  |
| N0+N1 | 25 | 27 |  |
| N2+N3 | 30 | 32 | 0.974 |
| Distant metastasis (M) status |  |  |  |
| M0 | 53 | 54 |  |
| M1 | 4 | 3 | 0.696 |
| TNM stage |  |  |  |
| I+II | 37 | 11 |  |
| III+IV | 20 | 46 | <0.001 |

^a^Mast cell number was acquired by counting CD45^+^CD117^+^FcεRI^+^ cells per million cells of tumor tissues. CEA, carcinoembryonic antigen; *H.pylori* Ab, *Helicobacter pylori* antibody.
