# Supplementary material for: Increased intratumoral mast cells foster immune suppression and gastric cancer progression through TNF-α-PD-L1 pathway
Source: J Immunother Cancer. 2019 Feb 26;7:54. doi: 10.1186/s40425-019-0530-3 (PMC6390584; doi:10.1186/s40425-019-0530-3)
Supplement: Supplementary file 13 — Table S6. Primer and probe sequences for real-time PCR analysis. (DOCX 20 kb) [file 40425_2019_530_MOESM13_ESM.docx]

**Supplementary Table 6.** Primer and probe sequences for real-time PCR analysis

| Gene | Primer of probe | Sequence 5′→3′ |
| --- | --- | --- |
| Human IFN-γ  Human Perforin 1  Human Granzyme B  Human GAPDH | forward  reverse  forward  reverse  forward  reverse  forward  reverse | AGTGATGGCTGAACTGTCGC  ACTGGGATGCTCTTCGACCT  GCTATCGTTAGTGCTAGTGGAT  ATCTGTCTGATGCGTATCCAAT  GAAAGTGCGAATCTGACTTACG  TTGTTTCGTCCATAGGAGACAA  ACCCAGAAGACTGTGGATGG  CAGTGAGCTTCCCGTTCAG |

For the probes, a FAM fluorescent reporter is coupled to the 5' end, and a TAMRA quencher is coupled to the 3'
end.
