# Supplementary material for: Increased intratumoral mast cells foster immune suppression and gastric cancer progression through TNF-α-PD-L1 pathway
Source: J Immunother Cancer. 2019 Feb 26;7:54. doi: 10.1186/s40425-019-0530-3 (PMC6390584; doi:10.1186/s40425-019-0530-3)
Supplement: Supplementary file 14 — Supplementary Materials and Methods. (DOCX 63 kb) [file 40425_2019_530_MOESM14_ESM.docx]

**Supplementary Materials and Methods**

**Mast cell and** **GC cell culture**

For culture of primary human umbilical cord blood-derived cultured mast cells (hCBMCs), human umbilical cord blood was obtained from obstetrics and gynecology department in the Southwest Hospital of Third Military Medical University. Umbilical cord blood mononuclear cells were isolated by density gradient centrifugation using Ficoll-Paque Plus. CD133^+^ cells were purified with CD133 microbeads. For the first 6 weeks, CD133^+^ cells were cultured in StemSpan Serum-Free Expansion Medium supplemented with penicillin (100 U/ml)/streptomycin (100 µg/ml), human recombinant (hr) stem cell factor (SCF) (100 ng/ml), hr IL-6 (50 ng/ml), hr IL-3 (30 ng/ml, only presents during the first 3 weeks). From week 6, fetal calf serum (FCS) (10%) was added into the culture medium. The generated mast cells were used for experiments until week 10. Mast cell purity was determined by surface staining of FcεRI and CD117, and toluidine blue staining.

The human mast cell line LAD2 (kindly provided by Dr. Wei Zhang, Shenzhen Key Laboratory for Translational Medicine of Dermatology, China) were cultured in Serum-Free Media (StemPro-34) supplemented with penicillin (100 U/ml)/streptomycin (100 µg/ml), hr SCF (100 ng/ml), hr IL-6 (50 ng/ml) and L-glutamine (2 mM) as described previously [1].

Human cell lines SGC-7901 (gastric cancer cell line) and HMC-1 (mast cell line) were purchased from China Center for Type Culture Collection (CCTCC, Wuhan, China) and cultured according to their guidelines.

**Flow cytometry**

Flow cytometric analysis was performed according to standard protocols. For intracellular cytokine measurements, the cells were stimulated for 5 hours with phorbol myristate acetate (50 ng/ml) plus ionomycin (1 μg/ml) in the presence of GolgiStop. Intracellular cytokine staining was performed after fixation and permeabilization using Perm/Wash solution. The cells were analyzed by multicolor flow cytometry with FACSCanto II (BD Biosciences). Data were analyzed with Flowjo software (TreeStar) or FACSDiva software (BD Biosciences).

**Western blot**

Western blot assays were performed on 10% SDS-PAGE gels using equivalent amounts of cell lysate proteins of samples. 5% BSA was used for blocking the PVDF membranes. Human p65, p-p65, p44/42, p-p44/42, JNK, p-JNK, p38, p-p38, AKT and p-AKT (ser473) were detected with their antibodies respectively. This was followed by incubation with horseradish peroxidase (HRP)-conjugated secondary antibodies. Bound proteins were visualized by using SuperSignal® West Dura Extended Duration Substrate kit.

**ELISA**

Human gastric tissues were collected, homogenized in 1 ml sterile Protein Extraction Reagent, and centrifuged. Tissue supernatants were collected for ELISA. Cell culture supernatants were collected as above for ELISA. Concentrations of IFN-γ in the mast cell-T cell co-culture supernatants, concentrations of CXCL12 and TNF-α in the tumor or non-tumor gastric tissues and TTCS or NTCS, and concentrations of IFN-γ, Perforin 1 and Granzyme B in the transplantation tumors or in the supernatants from the T cells cocultured with PD-L1-expressing mast cells were determined using ELISA kits according to the manufacturer’s instructions.

**Immunohistochemistry**

Paraformaldehyde-fixed and paraffin-embedded samples were cut into 5 µm sections. For immunohistochemical single-staining, the sections were incubated with mouse anti-human/mouse proliferating cell nuclear antigen (PCNA), rabbit anti-human CD3, or mouse anti-human tryptase antibodies respectively, either followed by HRP-conjugated rabbit anti-mouse IgG followed by diaminobenzidine. All the sections were finally counterstained with haematoxylin and examined using a microscope (Nikon Eclipse 80i; Nikon).

**Immunofluorescence**

Paraformaldehyde-fixed tumor tissue sections from GC patients or mast cell line (HMC-1 cells) were washed in PBS, blocked for 30 min with 20% goat serum in PBS, then stained for PD-L1, CXCR4, CD3, Ki-67 or TNFRII and tryptase, and CXCL12 or TNF-α and EpCam. Slides were examined with a confocal fluorescence microscope (LSM 510 META, Zeiss).

**Microarray experiments**

Gene expression profiles of human tumor tissues from GC patients were analyzed with the Affymetrix GeneChip Human Gene 1.0 ST Array (Affymetrix), strictly following the manufacturer’s protocol. Microarray experiments were performed at the Genminix Informatics (China) with the microarray service certified by Affymetrix.

**Real-time PCR**

Extracted RNA from tumor specimens were reverse-transcribed to cDNA by PrimeScriptTM RT reagent Kit. Real-time PCR was performed on the IQ5 (Bio-Rad) with the Real-time PCR Master Mix according to the manufacturer's specifications. Expression of IFN-γ, Perforin 1 and Granzyme B in the transplantation tumors was measured using the SYBR green method with the respective primers (Supplementary Table 6). The relative gene expression was expressed as fold change calculated by the ΔΔCt method.

**References:**

1. Radinger M, Jensen BM, Kuehn HS, Kirshenbaum A, Gilfillan AM. Generation, isolation, and maintenance of human mast cells and mast cell lines derived from peripheral blood or cord blood. Curr Protoc Immunol. 2010; Chapter 7:7-37.
